# Human Platelet Lysate as Alternative of Fetal Bovine Serum for Enhanced Human *In Vitro* Bone Resorption and Remodeling

**DOI:** 10.3389/fimmu.2022.915277

**Published:** 2022-06-20

**Authors:** Bregje W. M. de Wildt, Keita Ito, Sandra Hofmann

**Affiliations:** Orthopaedic Biomechanics, Department of Biomedical Engineering and Institute for Complex Molecular Systems (ICMS), Eindhoven University of Technology, Eindhoven, Netherlands

**Keywords:** *in vitro* model, co-culture, human platelet lysate, bone remodeling, 3Rs, osteoclasts

## Abstract

**Introduction:**

To study human physiological and pathological bone remodeling while addressing the principle of replacement, reduction and refinement of animal experiments (3Rs), human *in vitro* bone remodeling models are being developed. Despite increasing safety-, scientific-, and ethical concerns, fetal bovine serum (FBS), a nutritional medium supplement, is still routinely used in these models. To comply with the 3Rs and to improve the reproducibility of such *in vitro* models, xenogeneic-free medium supplements should be investigated. Human platelet lysate (hPL) might be a good alternative as it has been shown to accelerate osteogenic differentiation of mesenchymal stromal cells (MSCs) and improve subsequent mineralization. However, for a human *in vitro* bone model, hPL should also be able to adequately support osteoclastic differentiation and subsequent bone resorption. In addition, optimizing co-culture medium conditions in mono-cultures might lead to unequal stimulation of co-cultured cells.

**Methods:**

We compared supplementation with 10% FBS vs. 10%, 5%, and 2.5% hPL for osteoclast formation and resorption by human monocytes (MCs) in mono-culture and in co-culture with (osteogenically stimulated) human MSCs.

**Results and Discussion:**

Supplementation of hPL can lead to a less donor-dependent and more homogeneous osteoclastic differentiation of MCs when compared to supplementation with 10% FBS. In co-cultures, osteoclastic differentiation and resorption in the 10% FBS group was almost completely inhibited by MSCs, while the supplementation with hPL still allowed for resorption, mostly at low concentrations. The addition of hPL to osteogenically stimulated MSC mono- and MC-MSC co-cultures resulted in osteogenic differentiation and bone-like matrix formation, mostly at high concentrations.

**Conclusion:**

We conclude that hPL could support both osteoclastic differentiation of human MCs and osteogenic differentiation of human MSCs in mono- and in co-culture, and that this can be balanced by the hPL concentration. Thus, the use of hPL could limit the need for FBS, which is currently commonly accepted for *in vitro* bone remodeling models.

## 1 Introduction

Bone has multiple mechanical and metabolic functions that are maintained through lifelong remodeling by bone resorbing osteoclasts, bone forming osteoblasts, and regulating osteocytes. In the healthy situation, bone resorption and formation are mostly in balance, resulting in no net bone loss or gain. A shift in this balance, towards more formation or resorption, is a hallmark for pathologies like osteopetrosis or osteoporosis, respectively. Studies on these bone pathologies and development of drugs for their treatment are routinely performed in animal models. However, animal models represent human physiology insufficiently which is likely one of the reasons that only 9.6% of preclinically developed drugs are approved for regular clinical use (1, 2). Human *in vitro* models could enable the investigation of human healthy and pathological bone remodeling while addressing the principle of reduction, refinement, and replacement of animal experiments (3Rs) (3, 4). In this regard, osteoclast-osteoblast co-cultures have recently gained significant interest (5–8). In these co-cultures, human monocytes (MCs) and mesenchymal stromal cells (MSCs) are most frequently used as progenitor cells which are in culture differentiated into osteoclasts and osteoblasts (and eventually osteocytes), respectively (5). An advantage of using these progenitor cells is the possibility to personalize *in vitro* models (9).

Fetal bovine serum (FBS) is a culture medium supplement sourced from unborn calves at the slaughterhouse (10). FBS is currently easily available, relatively inexpensive, and it contains an excess of nutrients and proteins that support cell adhesion, growth, and proliferation. As a result, FBS is historically the most commonly used medium supplement for *in vitro* cultures, including for osteoclast-osteoblast co-cultures (5, 10). However, several safety, scientific, and ethical concerns against the use of FBS have been raised (10, 11). Batch to batch variation, zoonotic pathogens, and xenogeneic proteins that are incompatible with human physiology may cause undesired and irreproducible experimental results (10, 12, 13). In addition, with the aim to comply to the 3Rs, the use of animal components for *in vitro* alternatives to animal experiments is controversial (11). To overcome these concerns, human platelet lysate (hPL) has been suggested as a physiologically relevant alternative for FBS (11). Platelets contain a variety of proteins and nutrients that are vital for tissue regeneration (14–16), and may have an influence on healthy and pathological bone remodeling (17, 18). Their cargo can be released by lysis through *e.g.* freeze thaw cycles, sonification or platelet activation, resulting in platelet lysate.

**Figure 1:**
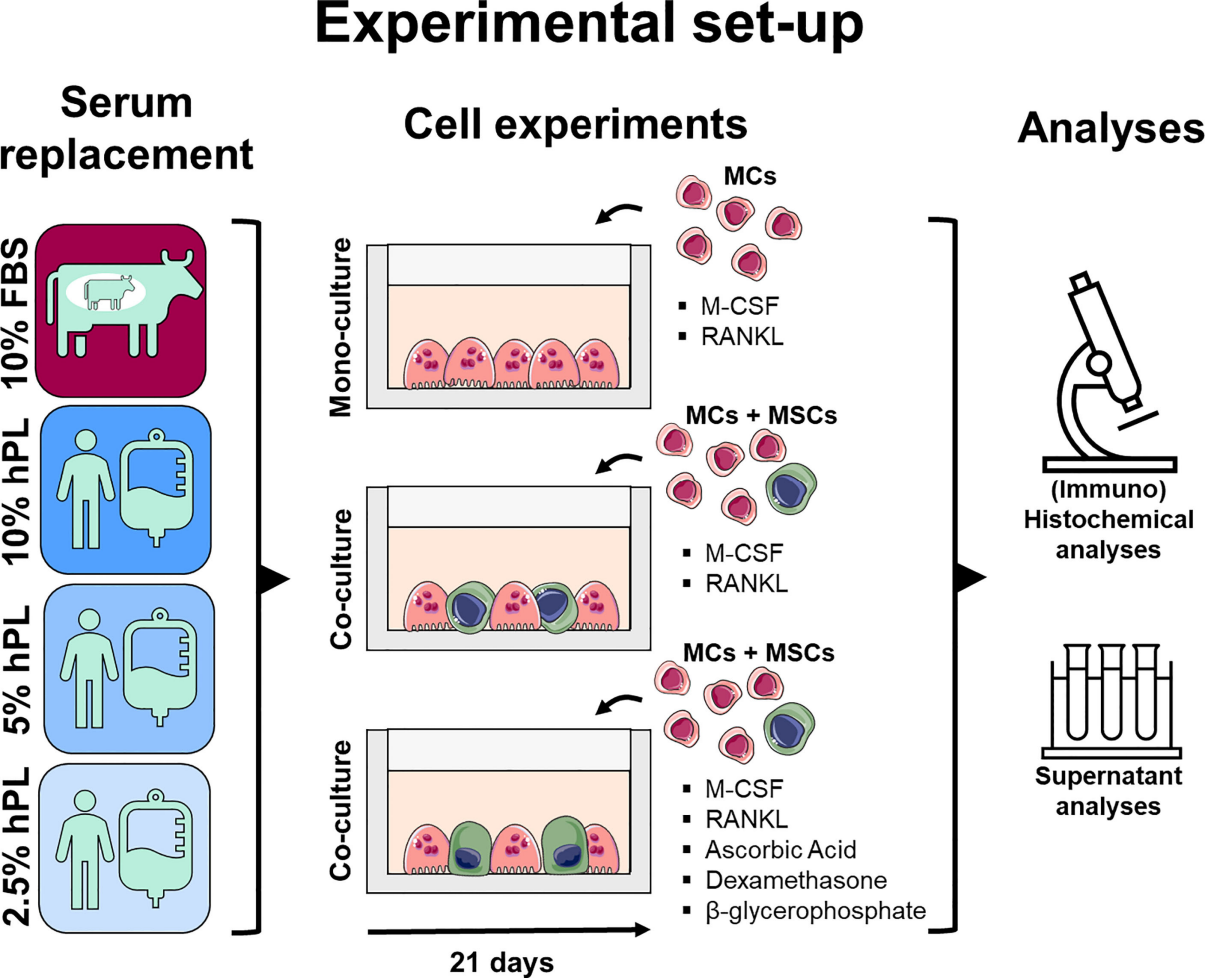
Medium supplementation with 10%, 5%, and 2.5% hPL were compared with 10% FBS for MC mono-cultures and (osteogenically stimulated) MC-MSC co-cultures. Cell cultures were analyzed using (immuno)histochemical and (immuno)cytochemical analyses to assess resorption, cell markers and cell morphology, and supernatant analyses to measure osteoclast activity and secreted RANKL and OPG. The figure was modified from Servier Medical Art, licensed under a Creative Common Attribution 3.0 Generic License (http://smart.servier.com/, accessed on 8 July 2021). fetal bovine serum (FBS), human platelet lysate (hPL), monocytes (MCs), mesenchymal stromal cells (MSCs), macrophage colony-stimulating factor (M-CSF), receptor activator of nuclear factor κB ligand (RANKL), osteoprotegerin (OPG).

While the use of hPL to replace FBS has been widely studied for human MSC cultures and osteogenic differentiation of these cells (19–26), the influence of hPL on osteoclastic differentiation of MCs is relatively unknown. Platelet-released supernatants could stimulate osteoclast differentiation and activity of human peripheral blood mononuclear cells (PBMCs) (27). This stimulatory effect was reduced by the presence of serum in the platelet-released supernatant (27). Recently, one study was published on the use of human serum and hPL as alternative of FBS for bone and cancer tissue models (28). They found an increase in Cathepsin K expression in human MC mono-cultures supplemented with 5% hPL. In contrast to cultures supplemented with 10% FBS and 10% human serum, they could not detect resorptive activity when measuring calcium concentration in the supernatant (28). Thus, there are indications that platelet-released factors and hPL could support osteoclastic differentiation of human PBMC and MC mono-cultures, but contradictory results are reported. Physiological bone remodeling is controlled by the direct and indirect interactions between osteoclasts and osteoblasts, with the receptor activator of nuclear factor κB ligand (RANKL)/osteoprotegerin (OPG) ratio as most important driver (29, 30). These interactions cannot be mimicked in mono-cultures. Thereby, co-culture medium should equally support both cell types to enable this interaction and to avoid unequal cell stimulation (3, 31). As such, studying the effect of hPL on MCs and MSCs and their differentiation in mono-cultures might be insufficient for the translation to *in vitro* bone remodeling models. Therefore, we investigated the use of three concentrations of hPL as a xenogeneic-free and physiologically relevant alternative for FBS on osteoclastic differentiation by MCs in mono-cultures. In addition, we explored the use of hPL for *in vitro* bone remodeling models by studying its influence on osteoclastic differentiation in MC-MSC co-cultures and osteogenically stimulated MC-MSC co-cultures (**Figure 1**).

## 2 Materials and Methods

In this study, medium supplementation with 10%, 5%, and 2.5% hPL were compared with 10% FBS for MC mono-cultures and (osteogenically stimulated) MC-MSC co-cultures. The tested concentrations were based on previous research. FBS is most frequently used in a concentration of 10% for MC-MSC co-cultures (5). For MSC expansion and osteogenic differentiation, 10% and 5% hPL are the most frequently used concentration (23, 24, 26). The 2.5% hPL concentration was added based on the hPL manufacturer’s advice for MC cultures. Cell cultures were analyzed using (immuno)histochemical and (immuno)cytochemical analyses to assess resorption, cell markers and cell morphology, and supernatant analyses to measure osteoclast activity and secreted RANKL and OPG.

### 2.1 Cell Culture

#### 2.1.1 MC Isolation

PBMCs were isolated from human peripheral blood buffy coats of three healthy donors (Sanquin, Eindhoven, The Netherlands; collected under their institutional guidelines and with informed consent per the Declaration of Helsinki). Buffy coats (~50 ml) were diluted with 0.6% w/v sodium citrate in phosphate buffered saline (citrate-PBS) until a final volume of 200 ml and layered per 25 ml on top of 10 ml Lymphoprep™ (07851, StemCell technologies, Köln, Germany) in 50 ml centrifugal tubes. After density gradient centrifugation (20 min at 800*x g*, lowest break), PBMCs were collected, resuspended in citrate-PBS, and washed four times in citrate-PBS supplemented with 0.01% bovine serum albumin (BSA, 10735086001, Sigma-Aldrich, Zwijndrecht, The Netherlands). PBMCs were frozen at 10^5^ cells/ml in freezing medium containing RPMI-1640 (RPMI, A10491, Thermo Fisher Scientific, Breda, The Netherlands), 20% FBS (BCBV7611, Sigma-Aldrich) and 10% dimethyl sulfoxide (DMSO, 1.02952.1000, VWR, Radnor, PA, USA) and stored in liquid nitrogen until further use. Before MC isolation, PBMCs were thawed, collected in medium containing RPMI, 10% FBS (BCBV7611, Sigma-Aldrich) and 1% penicillin-streptomycin (p/s, 15070063, Thermo Fisher Scientific), and after centrifugation resuspended in isolation buffer (0.5% w/v BSA in 2mM EDTA-PBS). MCs were enriched from PBMCs with manual magnetic activated cell separation (MACS) using the Pan Monocyte Isolation Kit (130-096-537, Miltenyi Biotec, Leiden, The Netherlands) and LS columns (130-042-401, Miltenyi Biotec) according to the manufacturer’s protocol, and directly used for experiments.

#### 2.1.2 MSC Isolation and Expansion

MSCs were isolated from human bone marrow (1M-125, Lonza, Walkersville, MD, USA; collected under their institutional guidelines and with informed consent) and characterized for surface markers and multilineage differentiation, as previously described (32). MSCs were frozen at passage 3 with 1.25*10^6^ cells/ml in freezing medium containing FBS (BCBV7611, Sigma-Aldrich) with 10% DMSO and stored in liquid nitrogen until further use. Before experiments, MSCs were thawed, collected in high glucose DMEM (hg-DMEM, 41966, Thermo Fisher Scientific), seeded at a density of 2.5*10^3^ cells/cm^2^ and expanded in medium containing hg-DMEM, 10% FBS (BCBV7611, Sigma-Aldrich), 1% Antibiotic Antimycotic (anti-anti, 15240, Thermo Fisher Scientific), 1% Non-Essential Amino Acids (11140, Thermo Fisher Scientific), and 1 ng/mL basic fibroblastic growth factor (bFGF, 100-18B, PeproTech, London, UK) at 37°C and 5% CO_2_. After 9 days, cells were detached using 0.25% trypsin-EDTA (25200, Thermo Fisher Scientific) and directly used for experiments at passage 4.

#### 2.1.3 MC Mono-Culture

For each donor, MCs were seeded in a culture plastic 96-wells plate and a osteo assay surface 96 wells plate (CLS3988, Corning, Amsterdam, The Netherlands) at a density of 9*10^4^ cells/well (3-4 repeats per donor, *N* = 9-12). MCs were cultured in priming medium containing α-MEM (41061, Thermo Fisher Scientific), 10% FBS (SFBS, Bovogen, East Keilor, Australia) or 10%, 5%, or 2.5% hPL (PE20612, PL BioScience, Aachen, Germany), 1% anti-anti, and 50 ng/ml macrophage colony-stimulating factor (M-CSF, 300-25, PeproTech). After 48 hours, priming medium was replaced by osteoclast medium (priming medium + 50 ng/ml RANKL (310-01, PeproTech) to induce osteoclastic differentiation. Cells were kept in culture for 21 days at 37°C and 5% CO_2_, medium was replaced 3x per week. Medium samples were collected and stored at -80°C on day 2, 7, 14 and 21 and culture photographs were taken (Invitrogen EVOS XL Digital Inverted Microscope).

#### 2.1.4 MC-MSC Co-Cultures

MCs of three donors were mixed with MSCs such that $\frac{1}{5}$ of the well area was covered by MSCs and $\frac{4}{5}$ by MCs. To accomplish this, MSCs and MCs were first mixed in the correct cell-ratio and then seeded together at a density of 2.5*10^3^ MSCs and 7.2*10^4^ MCs per well of a culture plastic 96-wells plate and a osteo assay surface 96 wells plate (3-4 repeats per donor, *N* = 9-12). By plating both cells in the same well, both direct and indirect cell-communication could be studied, which cannot be mimicked with trans-well systems. For MC-MSC co-cultures, cells were initially cultured in priming medium (Section 2.1.3) and after 48 hours, priming medium was replaced by osteoclast medium (Section 2.1.3) for the remaining culture period. For osteogenic MC-MSC co-cultures, cells were initially cultured in priming medium with osteogenic supplements (10 mM β-glycerophosphate (G9422, Sigma-Aldrich), 50 µg/ml ascorbic acid-2-phosphate (A8960, Sigma-Aldrich), and 100 nM Dexamethasone (D4902, Sigma-Aldrich)). After 48 hours, priming medium was replaced by osteoclast medium with osteogenic supplements for the remaining culture period. Cells of both co-cultures were kept in culture for 21 days at 37°C and 5% CO_2_, medium was replaced 3x per week. Medium samples were collected and stored at -80°C on day 2, 7, 14 and 21.

#### 2.1.5 Osteogenic MSC Mono-Culture

Osteogenic MSC mono-cultures (*N* = 4) were performed to compare the effect of FBS with hPL on osteogenic differentiation by MSCs and subsequent bone-like matrix production. The FBS and different concentrations of hPL were tested in an *in vitro* model for woven bone formation, as previously described (33). As the influence of hPL on osteogenic differentiation by MSCs has been frequently studied, we mainly focused on osteoclastic differentiation of MCs. Therefore, the details of this culture and its analyses can be found in the supplementary information.

### 2.2 Resorption Assay

To measure osteoclastic resorption after 21 days of culture, cells on Osteo Assay wells plates were removed by 5 min incubation with 5% bleach in ultra-pure water (UPW) and washed twice with UPW. As co-cultured cells were difficult to remove with the described treatment, cell remnants of co-cultures were mechanically removed by carefully scraping the wells with a pipet tip. To visualize the non-resorbed surface, osteo assay wells were stained with a modified Von Kossa. Briefly, wells were incubated with 5% w/v silver nitrate (209139, Sigma-Aldrich) in UPW for 30 min in the dark, washed with UPW, and incubated for 4 min with 5% w/v sodium carbonate (S7795, Sigma-Aldrich) in 3.7% neutral buffered formaldehyde. The staining solution was completely aspirated and plates were dried for 1 h at 50°C. To capture the entire well, tile scans were made with a bright field microscope (Zeiss Axio Observer Z1, 5x/0.13 EC Epiplan-Neofluar objective). Tile scans were stitched with Zen Blue software (version 3.1, Zeiss, Breda, The Netherlands). To enable segmentation and resorption quantification, scratches that were introduced by mechanical cell removal in co-cultures were manually masked whereafter image contrast was increased using Fiji (34). A clipping mask was created in Illustrator (Adobe Inc., San Jose, CA, USA) to remove the edges of the wells (**Figure S1**). Segmentation was performed in MATLAB (version 2019b, The MathWorks Inc., Natrick, MA, USA), using Otsu’s method for binarization with global thresholding, where the threshold was kept constant throughout the entire image (35). The total number of pixels within the well and the number of resorbed pixels were determined, such that the percentage resorbed area per well could be quantified.

### 2.3 Fluorescent Stainings

MC mono-cultures were stained with DAPI and Phalloidin to visualize cell nuclei and the actin cytoskeleton, respectively. In short, cells were fixed in 3.7% neutral buffered formaldehyde for 15 min, permeabilized in 0.5% triton X-100 in PBS for 10 min and blocked in 10% normal goat serum in PBS for 30 min. Cells were incubated with 0.1 µg/ml DAPI (D9542, Sigma-Aldrich) and 50 pmol Atto 647-conjugated Phalloidin (65906, Sigma-Aldrich) in PBS for 1 h. Images were taken with a confocal laser scanning microscope (Leica TCS SP8X, 20x/0.4 HC PL Fluotar L objective) at 2 different locations per well with 3 – 4 wells per donor. Images were subsequently deconvolved using the CLME deconvolution algorithm with the Batch Express function of Huygens Professional (version 20.04, Scientific Volume Imaging, The Netherlands). Cell morphology and cell area were extracted from the images using a custom-made pipeline in CellProfiler (version 4.04) (36). By using the actin cytoskeleton images, cells were segmented with the Minimum Cross-Entropy method with adaptive threshold. Cell eccentricity (as measure for morphology) and cell area were subsequently determined by CellProfiler (36).

To study the influence of MSCs on osteoclastic differentiation, MC-MSC co-cultures were stained with DAPI, Phalloidin, RANKL and OPG. Cell differentiation in MC-MSC co-cultures and osteogenic MC-MSC co-cultures was visualized with a staining for DAPI, Phalloidin, integrin-*β*3 as osteoclastic differentiation marker and runt-related transcription factor 2 (RUNX2) as osteoblastic differentiation marker. Briefly, one well per donor was fixed in 3.7% neutral buffered formaldehyde for 15 min, permeabilized in 0.5% triton X-100 in PBS for 10 min and blocked in 10% normal goat serum in PBS for 30 min. Primary antibodies were incubated overnight at 4 °C, secondary antibodies were incubated with 0.1 µg/ml DAPI and 50 pmol Atto 647-conjugated Phalloidin for 1 h at room temperature. Antibodies are listed in **Table S1**. Images were acquired with a confocal laser scanning microscope (Leica TCS SP8X, 20x/0.65 HC PL Apo CS2 objective). All images were prepared for presentation in Fiji (34).

### 2.4 Tartrate Resistant Acid Phosphatase Activity

Tartrate resistant acid phosphatase (TRAP) as a measure for osteoclastic differentiation was measured in cell supernatants. 10 µl supernatant or p-nitrophenol standard was incubated with 90 µl p-nitrophenyl phosphate buffer (1 mg/ml p-nitrophenyl phosphate disodium hexahydrate (71768, Sigma-Aldrich), 0.1 M sodium acetate, 0.1% triton X-100 and 30 µl/ml tartrate solution (3873, Sigma-Aldrich) in PBS) in 96-wells assay plates for 90 min at 37°C. To stop the reaction, 100 µl 0.3 M NaOH was added. Absorbance was read at 405 nm using a plate reader (Synergy™ HTX, Biotek) and absorbance values were converted to TRAP activity (converted p-nitrophenyl phosphate in nmol/ml/min) using standard curve absorbance values.

### 2.5 RANKL and OPG Quantification

Secreted RANKL and OPG were quantified in cell supernatants from day 7 of MC-MSC co-cultures with RANKL (ab213841, Abcam, Cambridge, UK) and OPG (EHTNFRSF11B, Thermo Fisher Scientific) enzyme-linked immunosorbent assays (ELISAs) according to the manufacturer’s protocols. To account for OPG and RANKL already present in FBS or hPL, FBS and hPL samples were included in the assays. To measure RANKL, samples were added to anti-human RANKL coated microwells. After 90 min incubation at 37°C, samples were replaced by biotinylated antibody solution followed by 60 min incubation at 37°C. After thorough washing, avidin-biotin-peroxidase complex (ABC) solution was added and plates were incubated for 30 min at 37°C. Wells were again washed and color developing agent was added followed by 15 min incubation in the dark at 37°C. To stop the reaction, stop solution was added and absorbance was measured at 450 nm in a plate reader. To measure OPG, samples were added to anti-human OPG coated microwells and incubated for 2.5 h at room temperature with gentle shaking. Wells were subsequently washed, biotinylated antibody solution was added followed by 60 min incubation at room temperature with gentle shaking. After washing, streptavidin-HRP solution was added and incubated in the wells for 45 min with gentle shaking. Wells were subsequently washed and incubated with substrate solution for 30 min in the dark with gentle shaking. The enzymatic reaction was stopped with stop solution and absorbance was measured at 450 nm in a plate reader. All absorbance values were converted to RANKL and OPG concentrations using standard curve absorbance values.

### 2.6 Supplement Characterization

#### 2.6.1 Total Protein Measurement

To quantify total protein content in FBS and hPL, a bicinchoninic acid (BCA) assay (23225, Thermo Fisher Scientific) was performed according to the manufacturer’s instructions. In short, 200 µl BCA working reagent was added to 25 µl sample in 96-wells assay plates, followed by 30 sec mixing on a plate shaker and 30 min incubation at 37 °C. The assay plate was then cooled to room temperature and absorbance was measured at 562 nm on a plate reader. Absorbance values were converted to protein concentrations using standard curve absorbance values.

#### 2.6.2 Alkaline Phosphatase and Tartrate Resistant Phosphatase Activity

As mineralization can be directly influenced by serum alkaline phosphatase (ALP) activity (37), ALP and TRAP activity were measured in FBS and hPL. TRAP activity was measured as described in section 2.4. ALP activity was determined by adding 20 µl of 0.75 M 2-amino-2-methyl-1-propanol (A65182, Sigma-Aldrich) to 80 µl sample in 96-wells assay plates. Subsequently, 100 µl substrate solution (10 mM p-nitrophenyl-phosphate (71768, Sigma-Aldrich) in 0.75 M 2-amino-2-methyl-1-propanol) was added and wells were incubated at room temperature for 15 minutes. To stop the reaction, 100 µl 0.2 M NaOH was added. Absorbance was measured with a plate reader at 450 nm and these values were converted to ALP activity (converted p-nitrophenyl phosphate in µmol/ml/min) using standard curve absorbance values.

#### 2.6.3 Calcium Measurement

Since extracellular calcium could influence osteoclast attachment and osteoclastic resorption (38), a calcium assay (Stanbio, 0150-250, Block Scientific, Bellport, NY, USA) was performed to measure calcium concentration in FBS and hPL, according to the manufacturer’s instructions. Briefly, 95 µl Cresolphthalein complexone reaction mixture was added to 5 µl sample and incubated at room temperature for 1 min. Absorbance was measured at 550 nm with a plate reader and absorbance values were converted to calcium concentrations using standard curve absorbance values.

#### 2.6.4 Phosphate Measurement

As high extracellular phosphate levels could inhibit osteoclast differentiation (39, 40), phosphate concentration in FBS and hPL was measured using a Malachite Green phosphate assay (MAK307, Sigma-Aldrich). According to the manufacturer’s instructions, 80 µl of sample was mixed with 20 µl of working reagent in 96-wells assay plates. Wells were incubated for 30 min at room temperature and absorbance was subsequently measured at 620 nm using a plate reader. Absorbance values were converted to phosphate concentrations using standard curve absorbance values.

#### 2.6.5 Multiplex Immunoassays

To explore the protein content of hPL, a total of 21 proteins that have been reported to influence bone resorption, formation or remodeling were quantified using multiplex immunoassays at the Multiplex Core Facility (MCF) of the Laboratory for Translational Immunology of the University Medical Center Utrecht, the Netherlands. Immunoassays were developed and validated by the MCF and based on Luminex xMap technology (Luminex, Austin, TX, USA) (41). In short, hPL was incubated with MagPlex microspheres (Luminex) for 1 h at room temperature with continuous shaking, followed by 1 h incubation with biotinylated antibodies and 10 min incubation with phycoerythrin-conjugated streptavidin in high performance ELISA buffer (HPE, Sanquin, Hambug, Germany). Data acquisition was performed with FLEXMAP 3D equipment in combination with xPONENT software (version 4.3, Luminex), and analyzed by 5-parametric curve fitting using Bio-Plex Manager software.

### 2.7 Statistical Analyses

Statistical analyses were performed and graphs were prepared in GraphPad Prism (version 9.3.0, GraphPad, La Jolla, CA, USA) and R (version 4.1.2) (42). Data were tested for normality in distributions and equal variances using Shapiro-Wilk tests and Levene’s tests, respectively. When these assumptions were met, mean ± standard deviation are presented, and to test for differences, an independent t-test (for the comparison in phosphatase activity and protein, phosphate, and calcium concentration between FBS and hPL), one-way ANOVA (for RANKL and OPG data), or two-way ANOVA (for TRAP data) were performed followed by Turkey’s *post hoc* tests with adjusted p-value for multiple comparisons. Other data are presented as median ± interquartile range and were tested for differences with the non-parametric Kruskal-Wallis test with Dunn’s *post hoc* tests with adjusted p-value for multiple comparisons. With a *p-*value of <0.05 differences were considered statistically significant.

## 3 Results

### 3.1 HPL Outperforms FBS for Osteoclast Differentiation and Resorption in MC Mono-Cultures

**Figure 2:**
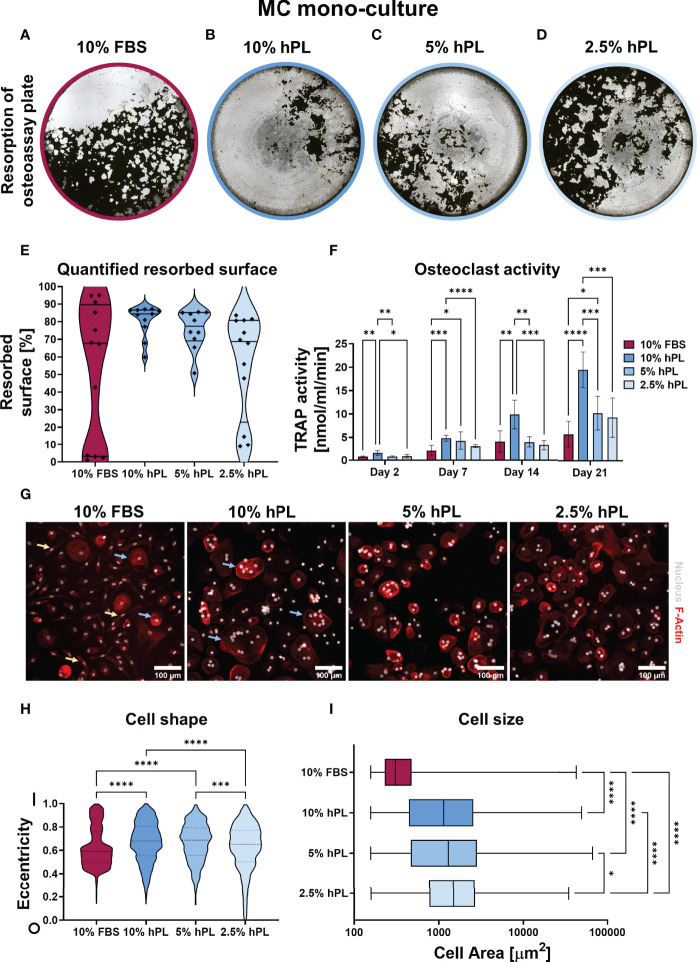
**(A–D)** Von Kossa stained decellularized resorption plates of MC mono-cultures. **(E)** Quantification of resorbed area, *p* < 0.05 (Kruskal-Wallis). **(F)** TRAP activity quantification, *p* < 0.05 for culture time, culture condition and their interaction (two-way ANOVA and Turkey’s *post hoc* tests within each time point). **(G)** Micrographs of MCs stimulated to undergo osteoclastic differentiation, stained for F-Actin (red) and the nucleus (gray). **(H)** Quantification of cell shape, 0 indicates a perfect circle while 1 indicates a line, *p* < 0.05 (Kruskal-Wallis and Dunn’s *post hoc* tests). **(I)** Quantification of cell size, *p* < 0.05 (Kruskal-Wallis and Dunn’s *post hoc* tests). Asterisks in figures represent results of *post hoc* analyses (*p < 0.05, **p < 0.01, ***p < 0.001, ****p < 0.0001). fetal bovine serum (FBS), human platelet lysate (hPL), monocytes (MCs), tartrate resistant acid phosphatase (TRAP).

After 21 days culture with different concentrations of hPL and 10% FBS, decellularized and Von Kossa stained osteo-assay plates of MC mono-cultures cultured with hPL showed more resorption than MCs cultured with FBS (**Figures 2A–D**). Resorption under influence of hPL seemed to have a dose dependent response, with most resorption in MCs cultured with 10% hPL and least resorption when cultured with 2.5% hPL. Quantification after segmentation of these osteo-assay plates revealed a significantly different resorbed area under influence of different serum supplements and a high variation in MCs cultured with FBS (67.7% ± 86.5%) when compared to MCs cultured with 10% and 5% hPL (84.4% ± 9.50% and 77.4% ± 16.12%, respectively) (**Figure 2E**). This high variation might indicate a donor-dependent response of osteoclastic differentiated MCs to 10% FBS. TRAP activity measurements supported the resorption results, with highest TRAP activity for cells cultured with 10% hPL which differed significantly with TRAP activity for cells cultured with 10% FBS at all time points (**Figure 2F**). TRAP activity increased over the entire culture duration over all conditions but seemed to increase more for MCs cultured with hPL towards day 21. At day 21, TRAP activity of both 10% and 5% hPL groups was significantly higher than TRAP activity in the 10% FBS group. When looking at cell morphology, cells cultured under influence of hPL showed typical osteoclast characteristics like an actin ring and multiple nuclei (**Figure 2G**). In wells cultured with FBS, a more heterogeneous cell population was found, including both osteoclastic cells and spindle shaped cells (**Figure 2G**, blue arrows and yellow arrows, respectively). Quantification of cell shapes, expressed as eccentricity, found in all processed images revealed indeed two different cell morphologies for cells cultured with FBS: cells with an eccentricity value close to 1, having a longitudinal possibly macrophage-like morphology, and cells with an eccentricity value around 0.5, which are more rounded and possibly indicative for the osteoclast-like cells (**Figure 2H**). This morphology significantly differed from the cell morphology in wells that were cultured with 10% and 5% hPL, which showed a more normal distribution of cell eccentricity. Besides the difference in cell shape, cell size, which is associated with osteoclast functionality (43), was also significantly lower in cells cultured with FBS compared to cells cultured with hPL (**Figure 2I**). Interestingly, cells cultured with 2.5% hPL showed a significantly rounder morphology and were significantly bigger than cells cultured with 10% and 5% hPL. As osteoclasts have a life-span of approximately 2-3 weeks (44, 45), MCs that have undergone quick osteoclastic differentiation, which is potentially the case in the 10% hPL group, could have released the well surface already before fixation at day 21, possibly affecting some results. This seems likely the case judging from culture photographs of day 18 (**Figure S2**).

### 3.2 MSCs Reverse the hPL Dose-Dependent Resorptive Activity of Osteoclasts

**Figure 3:**
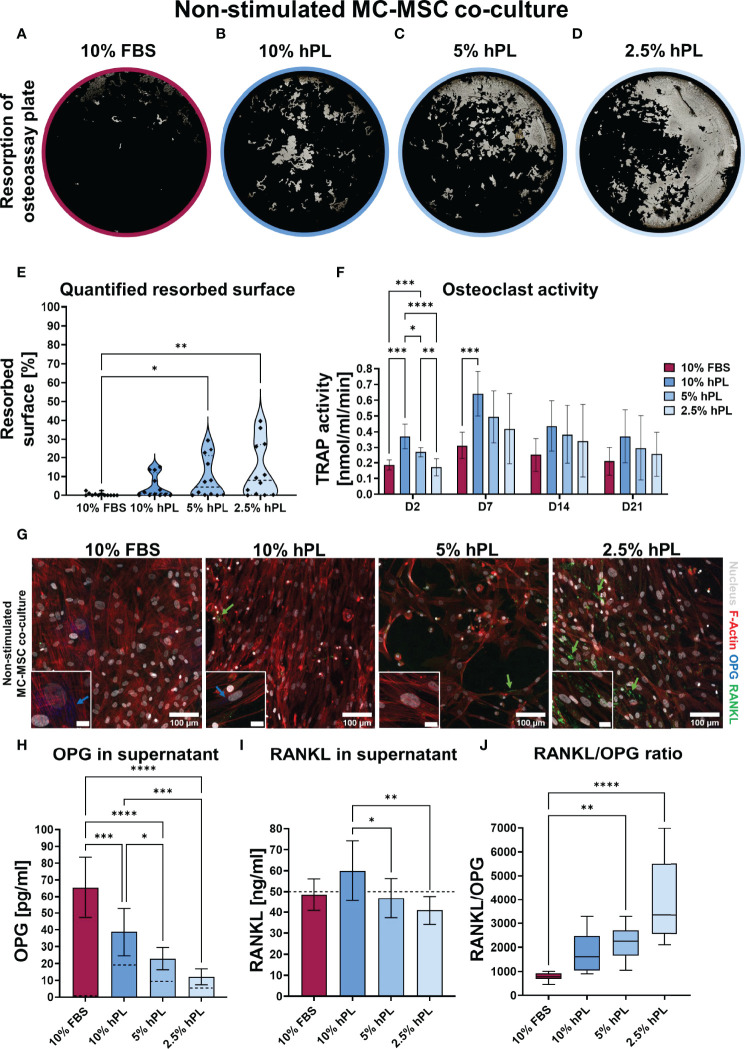
**(A–D)** Von Kossa stained decellularized resorption plates of MC-MSC co-cultures. **(E)** Quantification of resorbed area, *p*<0.05 (Kruskal-Wallis and Dunn’s *post hoc* tests). **(F)** TRAP activity quantification, *p* < 0.05 for culture time, culture condition, no interaction effect (two-way ANOVA and Turkey’s *post hoc* tests within each time point). **(G)** Micrographs of MC-MSC co-cultures stimulated to undergo osteoclastic differentiation, stained for F-Actin (red), the nucleus (gray), OPG (blue) and RANKL (green). Images were taken at locations rich of MSCs to detect their influence on OPG and RANKL production. Scale bar in insert is 20 µm. **(H)** Quantification of OPG in supernatant, dashed lines represents concentration in the medium control, *p* < 0.05 (one-way ANOVA and Turkey’s *post hoc* tests). **(I)** Quantification of RANKL in supernatant, dashed line represents concentration exogenous RANKL added to the medium, *p* < 0.05 (one-way ANOVA and Turkey’s *post hoc* tests). **(J)** RANKL/OPG ratio in supernatant, *p* < 0.05 (Kruskal-Wallis and Dunn’s *post hoc* tests). Asterisks in figures represent results of *post hoc* analyses (*p < 0.05, **p < 0.01, ***p < 0.001, ****p < 0.0001). fetal bovine serum (FBS), human platelet lysate (hPL), monocytes (MCs), mesenchymal stromal cells (MSCs), tartrate resistant acid phosphatase (TRAP), osteoprotegerin (OPG), receptor activator of nuclear factor κB ligand (RANKL).

After 21 days of MC co-culture with MSCs, again most resorption was present in groups cultured with hPL when compared to cells cultured with 10% FBS (**Figures 3A–D**). However, opposite to the MC mono-culture, a reversed dose-dependent relationship was found between hPL concentration and resorbed area (**Figures 3B–E**). While in mono-culture resorption was highest in 10% hPL, in co-culture with MSCs resorption was highest in 2.5% hPL. Quantification after segmentation of the resorbed area confirmed significantly more resorption in wells cultured with 5% and 2.5% hPL when compared to 10% FBS (**Figure 3E**). In addition, while MC mono-cultures showed an increase in TRAP activity over time, in co-culture with MSCs TRAP activity initially increased but decreased again after day 7 (**Figure 3F**). No different TRAP activity between groups could be found on day 14 and day 21 of culture. This indicates an inhibitory effect of MSCs on osteoclastic differentiation of MCs. As MSCs could influence osteoclastic differentiation by the RANKL/OPG ratio (29), these factors were visualized in the cell cultures and quantified in culture supernatants. Immunocytochemical staining revealed some MSCs expressing OPG in wells cultured with FBS and 10% hPL (**Figure 3G**). In contrast, RANKL was mostly present on the cell surface of MSCs cultured with 2.5% hPL (**Figure 3G**). Quantification of OPG in the medium on day 7 showed the same trend, with significantly more OPG production in wells cultured with FBS than in wells cultured with hPL at any of the concentrations (**Figure 3H**). Of importance, as OPG could not be measured in FBS, all OPG measured in the cell supernatant is a result of OPG production by the cells, while for hPL, some OPG measured could already be explained by the OPG concentration measured in hPL (**Figure 3H**, dashed lines in bars). The RANKL concentration that was measured in the cell supernatant could mainly be explained by the used concentration in the cell culture medium (50 ng/ml) (**Figure 3I**). Only in wells cultured with 10% hPL additional RANKL seemed to be produced by the cells, which resulted in a significantly higher RANKL concentration than in wells cultured with 5% or 2.5% hPL. Overall, the RANKL/OPG ratio measured in cell supernatants was highest in wells cultured with 2.5% hPL (**Figure 3J**), which makes this concentration probably the most potent inducer of osteoclastic differentiation in co-culture with MSCs.

### 3.3 Osteogenic Stimulation of MSCs Impedes Osteoclast Motility Independent From hPL Dose

**Figure 4:**
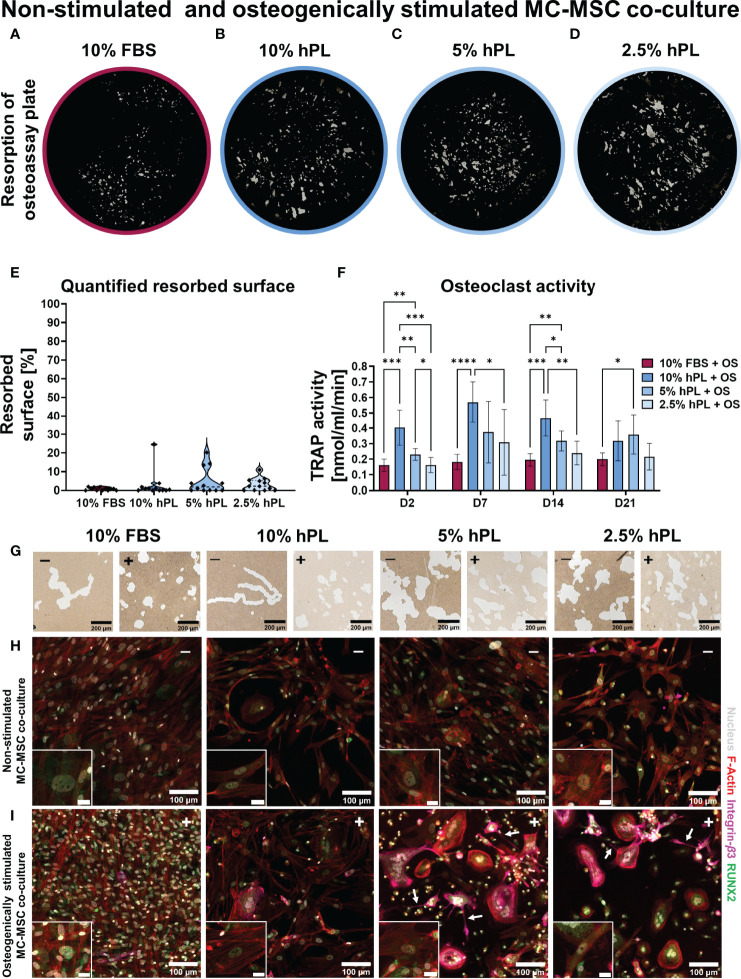
**(A–D)** Von Kossa stained decellularized resorption plates of osteogenically stimulated MC-MSC co-cultures. **(E)** Quantification of resorbed area, *p*=ns (Kruskal-Wallis test). **(F)** TRAP activity quantification, *p*<0.05 for culture time, culture condition and their interaction (two-way ANOVA and Turkey’s *post hoc* tests within each time point). **(G)** Micrographs of osteo assay plates indicating resorption trenches and resorption pits of non-stimulated co-cultures (-) and osteogenically stimulated co-cultures (+). **(H)** Micrographs of non-stimulated MC-MSC co-cultures, and **(I)** osteogenically stimulated MC-MSC co-cultures, stained for F-Actin (red) the nucleus (gray), Integrin-*β*3 (pink) and RUNX2 (green). Scale bar in insert is 20 µm. Asterisks in figures represent results of *post hoc* analyses (*p < 0.05, **p < 0.01, ***p < 0.001, ****p < 0.0001). fetal bovine serum (FBS), human platelet lysate (hPL), monocytes (MCs), mesenchymal stromal cells (MSCs), tartrate resistant acid phosphatase (TRAP), osteogenic supplements (OS), runt-related transcription factor 2 (RUNX2).

When stimulating MC-MSC co-cultures with osteogenic factors added to the medium, 10% FBS again seemed to induce the least resorption (**Figures 4A–D**). Quantification after segmentation of the resorbed area revealed indeed least resorption in wells cultured with FBS, although not statistically significant (**Figure 4E**). Resorption in groups cultured with hPL also seemed limited and the resorbed area featured a different shape from wells cultured without osteogenic supplements (**Figure 4G**). Osteoclasts in non-stimulated MC-MSC co-cultures formed resorption trenches in the osteo assay surface, whereas osteoclasts in osteogenically stimulated MC-MSC co-cultures formed resorption pits (46). Similar to the MC-MSC co-cultures, osteogenically stimulated MC-MSC co-cultures showed an initial increase in TRAP activity, followed by a decrease after day 7 (**Figure 4F**). After 21 days of culture, no differences in TRAP activity were found between the different concentrations of hPL. It is expected that OPG and RANKL also played a role in the inhibition of osteoclastic differentiation in osteogenically stimulated MC-MSC co-cultures, although no clear differences between groups were found after immunocytochemical staining of OPG and RANKL (**Figure S3**). Osteogenically stimulated MSC mono-cultures indeed showed a similar trend as in MC-MSC co-cultures, with highest OPG concentration in cells cultured with 10% FBS (69.9 ± 4.07 pg/ml) and lowest in cells cultured with 2.5% hPL (23.5 ± 3.81 pg/ml) (**Figure S4**). Secreted RANKL could only be detected in osteogenically stimulated MSCs cultured with 10% hPL (**Figure S4**). To investigate the influence of osteogenic stimulation on MC and MSC differentiation, cells were stained for differentiation markers RUNX2 [osteogenic transcription factor (47)] and Integrin-*β*3 [mature osteoclast marker (48)]. Osteogenic differentiation was confirmed by the presence of nuclear RUNX2 in all groups cultured with osteogenic supplements (**Figure 4I**). MSCs cultured with 10% FBS and osteogenic supplements showed most clear nuclear RUNX2 expression (**Figure 4I**). FBS seems to be superior to hPL for osteogenic differentiation, as confirmed by the abundant presence of collagen, nuclear RUNX2, osteopontin, ALP and mineralization in osteogenically stimulated MSC mono-cultures (Figure S4). Surprisingly, also non-stimulated MSCs in MC-MSC co-cultures expressed nuclear RUNX2, with most clear presence in nuclei of cells cultured with hPL (**Figure 4H**). RUNX2 was also observed in the cytoplasm of MCs and osteoclasts, which has recently been discovered and suggested to promote osteoclastic differentiation (49). A clear difference between osteogenically stimulated and non-stimulated co-cultures was found for the Integrin-*β*3 staining. Integrin-*β*3 was mainly present in osteoclasts cultured with osteogenically stimulated MSCs (**Figure 4I**). These integrin-*β*3 positive cells also showed cell processes that were not observed in MC mono-cultures or non-stimulated MC-MSC co-cultures (**Figure 4I**, white arrows).

### 3.4 Supplement Characterization

**Figure 5:**
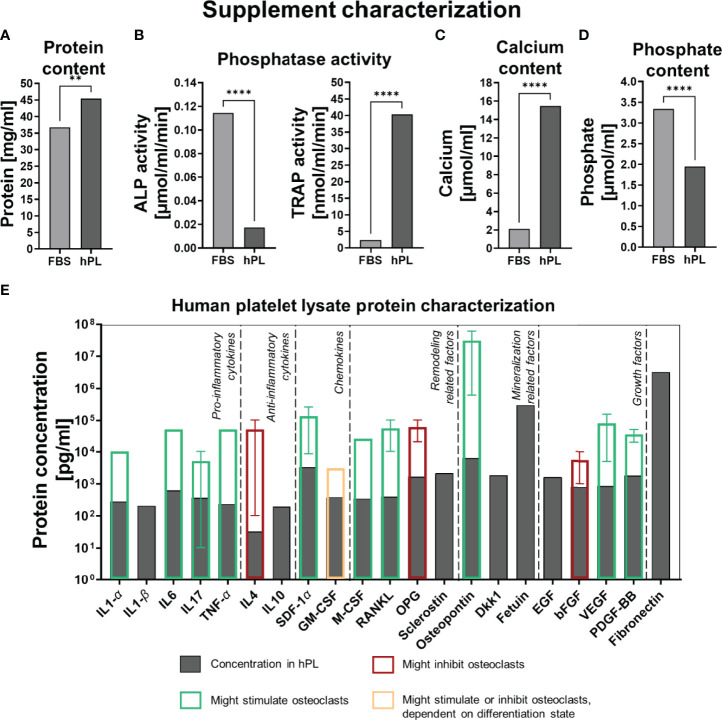
**(A)** Protein concentration in FBS and hPL, *p*<0.05 (independent t-test). **(B)** Phosphatase activity (ALP and TRAP) in FBS and hPL, *p* < 0.05 (independent t-test). **(C)** Calcium concentration in FBS and hPL, *p* < 0.05 (independent t-test). **(D)** Phosphate concentration in FBS and hPL, *p* < 0.05 (independent t-test). Asterisks indicate significant differences (**p < 0.01, ****p < 0.0001). **(E)** Quantification of bone and bone-remodeling related factors in hPL. Transparent bars indicate the mean effective concentration found by *in vitro* experiments from literature, error bars represent minimal and maximal reported effective concentration, border color represents the direction of the found effect. fetal bovine serum (FBS), human platelet lysate (hPL), alkaline phosphate (ALP), tartrate resistant acid phosphatase (TRAP), interleukin (IL), tumor necrosis factor (TNF), stromal derived factor (SDF), granulocyte-macrophage colony-stimulating factor (GM-CSF), macrophage colony-stimulating factor (M-CSF), receptor activator of nuclear factor κB ligand (RANKL), osteoprotegerin (OPG), Dickkopf WNT Signaling Pathway Inhibitor 1 (Dkk-1), epidermal growth factor (EGF), basic fibroblastic growth factor (bFGF), vascular endothelial growth factor (VEGF), platelet derived growth factor-BB (PDGF-BB).

To explore the differences between cells cultured with FBS or hPL found in this study, we attempted to characterize some components of the hPL used. For hPL, a higher protein concentration (45.4 mg/ml) was measured compared to FBS (36.7 mg/ml) (**Figure 5A**). Interestingly, quantification of ALP as mineralization related phosphatase, and TRAP as resorption related phosphatase, revealed a clear difference between FBS and hPL (**Figure 5B**). Whereas a significantly higher ALP activity was measured in FBS than in hPL, TRAP activity was significantly higher in hPL. With Luminex, the concentration of 21 proteins that have been reported to influence bone remodeling, were quantified (**Figure 5E**) and compared to effective concentrations used for *in vitro* studies related to bone remodeling (**Table S2**). As a result, relative high concentrations of pro-inflammatory cytokines were measured (interleukin (IL) 1-*α*: 284 pg/ml, IL1-*β*: 208 pg/ml, IL6: 624 pg/ml, IL17: 370 pg/ml and tumor necrosis factor-*α* (TNF-*α*): 237 pg/ml), when compared to the concentration of anti-inflammatory cytokines (IL4: 32.7 pg/ml and IL10: 197 pg/ml) (**Figure 5E**). Based on *in vitro* studies from literature, most of these factors could only have had an effect on osteoclast differentiation and resorption at higher concentrations (**Table S2**). Only the IL17 concentration measured in hPL was within the range of reported effective *in vitro* concentrations on osteoclast for the 10% and 5% hPL groups. In addition, the important proteins for osteoclast adhesion, osteopontin and fibronectin, were detected in hPL (2.15 ng/ml and 3.21 µg/ml, respectively). As expected, hPL also contained growth factors typical for platelets which might have affected osteoclastic differentiation and resorption (epidermal growth factor (EGF): 1.62 ng/ml, basic fibroblastic growth factor (bFGF): 812 pg/ml, vascular endothelial growth factor (VEGF): 869 pg/ml and platelet derived growth factor-BB (PDGF-BB): 8.16 ng/ml). One discrepancy was found in our data. The OPG concentration in hPL was measured with Luminex and ELISA, but Luminex gave a much higher concentration (193 pg/ml for ELISA compared to 1.69 ng/ml for Luminex). Quantification of calcium concentrations revealed a higher calcium concentration for hPL (15.5 µmol/ml) than for FBS (2.11 µmol/ml), which is for 10% hPL comparable to the for osteoclastogenesis most optimal calcium concentration found in literature (10% hPL: ~1.55 µmol/ml, most effective concentration in literature: 1.2 µmol/ml (38)) (**Figure 5C**). For phosphate, a higher concentration was found in FBS (3.34 µmol/ml) when compared to hPL (1.95 µmol/ml) (**Figure 5D**). However, for both medium supplements this was below the range of effective concentrations found in literature (**Table S2**).

## 4 Discussion

Although alternatives are available, FBS is still the most commonly used culture medium supplement. However, its use is increasingly considered controversial due to several safety, scientific and ethical concerns (10, 11). Also, for osteoclast-osteoblast co-cultures with human MCs and MSCs as progenitors, aiming at mimicking human bone remodeling and related pathologies *in vitro¸* FBS is still the common standard (5). hPL has been suggested as alternative for FBS and is already frequently studied for MSC propagation and osteogenic differentiation of these cells (19–26). In contrast, the effect of hPL on osteoclastic differentiation of MCs is relatively unknown. Therefore, the aim of this study was to investigate the influence of hPL as culture medium supplement at concentrations of 10%, 5% and 2.5% on osteoclastic differentiation and resorption of human MCs, using 10% FBS as a control. In addition, as physiological bone resorption is partly regulated by cells of the MSC-osteoblast-osteocyte lineage with the RANKL/OPG ratio, the influence of hPL at different concentrations on osteoclastic differentiation and resorption was also investigated under influence of (osteogenically stimulated) MSCs.

We initially studied the potential of hPL to support osteoclastic differentiation in MC mono-cultures. As a result, osteoclastic differentiation and resorption could be enhanced when using hPL instead of FBS. Whereas hPL induced MCs to undergo homogeneous osteoclastic differentiation followed by resorption consistent for all donors and hPL concentrations, MCs’ response to FBS seemed less reproducible. More specifically, FBS induced a heterogeneous cell population and differences in resorptive activity between different donors. The differences in resorptive activity between different donors was not reflected in the variation in TRAP activity measured in the cell supernatant, as observed before (50). The spindle shaped cells in MC mono-cultures supplemented with 10% FBS could indicate differentiation towards macrophages, a phenomenon that has been reported previously for MCs cultured in FBS and stimulated to undergo osteoclastic differentiation (28). Human MCs can polarize into macrophage phenotypes including the more pro-inflammatory M1 and the more anabolic M2 macrophages with both different roles in tissue regeneration and remodeling (51). Villasante et al. (2021) found more elongated cells in MCs treated with FBS when compared to MCs treated with human serum and hPL (28). These elongated cells were characterized as M2 macrophages based on their shape and gene expression profile (28, 52). Human serum induced M1 polarization which could have enabled further differentiation towards osteoclasts (28, 53). As platelets are activated upon tissue damage likely stimulating M1 polarization, hPL might have positively influenced osteoclast formation by precursor differentiation (53). In co-culture with MSCs, hPL indeed induced M1 polarization in MC derived macrophages (31). This hypothesis would need further characterization of cell markers and secretory profiles at multiple time points during culture. Although this is a limitation of the current study, future studies would benefit from including analyses on macrophage polarization in addition to analyses on osteoclast differentiation. Furthermore, it is recommended that future studies aim to elucidate the molecular mechanisms involved in the osteoclastic differentiation process under influence of hPL, using for example gene expression analyses.

Next, the influence of 10%, 5%, and 2.5% hPL and 10% FBS on osteoclastic differentiation and resorption in co-culture with MSCs and osteogenically stimulated MSCs was investigated. MCs/osteoclasts and MSCs/osteoblasts both have their preferred medium that supports their growth and function but that could inhibit the co-cultured cells in osteoclast-osteoblast co-cultures (3, 5). As such, supporting only osteoclasts could lead to imbalanced or pathological remodeling as for example in osteoporosis (6). Therefore, culture medium for “physiological” or “healthy” *in vitro* bone remodeling models should be carefully developed and tested such that it supports the balanced function of both osteoclasts and osteoblasts (5). The reversed dose-dependent effect that was found in MCs co-cultured with non-stimulated MSCs, when compared to MC mono-cultures, underlines the importance of developing co-culture medium using co-cultures. While hPL again outperformed 10% FBS for osteoclast differentiation and resorption, 2.5% hPL induced most resorption in MC-MSC co-cultures. The secreted RANKL/OPG ratio, as a major predictor for osteoclastogenesis, was indeed highest in cells cultured with 2.5% hPL and lowest in cells cultured with 10% FBS. FBS seemed to inhibit osteoclastic differentiation and resorption almost completely in MC-MSC co-cultures. Recently Tylek et al. (2019) found a shift towards MSCs being the most prominent cell type in macrophage-MSC co-cultures under influence of FBS supplementation, while hPL equally supported both cell-types in terms of attachment and proliferation (31). As mainly MSCs were observed in (osteogenically stimulated) MC-MSC co-cultures, FBS might have supported mainly MSCs. In line with this observation, FBS also seemed to induce best the osteogenic differentiation of MSCs as detected by most prominent nuclear RUNX2 in osteogenically stimulated MC-MSC co-cultures. MSC mono-cultures supported this finding with highest ALP activity, most mineralization, and most prominent collagen formation when constructs were cultured with 10% FBS. In these mono-cultures, OPG measurements followed the same trend as ALP activity, mineralization and collagen formation observations. This is in line with the hypothesis that during the bone formation phase of the remodeling cycle, osteoclastic resorption is inhibited by osteoblasts through the RANKL/OPG ratio as resorption is not desired anymore (3). Taken together, 10% FBS seemed to unequally support MCs and MSCs when co-cultured. More specifically, FBS induced the most prominent effect on MSCs and their osteogenic differentiation while hPL supported both osteogenic and osteoclastic differentiation. As we only studied osteogenic differentiation in MC-MSC co-cultures by localizing RUNX2 expression, future studies could further elucidate the influence of FBS and hPL on osteogenic differentiation in MC-MSC co-cultures as *in vitro* remodeling models.

Interestingly, osteogenic stimulation of MC-MSC co-cultures induced differences in resorption patterns on osteo assay surfaces when compared to non-stimulated MC-MSC co-cultures. Instead of resorption trenches, osteogenic stimulation led to the formation of resorption pits. This phenomenon was observed in both FBS and hPL treated groups, indicating an effect of the osteogenic supplements. The difference in resorption trenches and pits has been described as a result of insufficient collagen degradation by cathepsin K, impeding osteoclasts’ motility which results in a pit instead of a trench (46). The formation of these trenches has been described for remodeling pathologies like osteoporosis and might thus be undesirable for “healthy” *in vitro* bone remodeling models (46). It is unclear whether the pits found in this study are a result of collagen production by osteogenically differentiated MSCs, or a difference in cathepsin K production by osteoclasts in the different co-cultures. While this is a limitation of the current study, it would be interesting for future research to evaluate cathepsin K production and collagen formation and degradation markers like c-propeptide of type I procollagen (CICP) and c-terminal telopeptide of type-I collagen (CTX), respectively (5). In these osteogenically stimulated co-cultures, integrin-*β*3 and extended cell processes were also observed which could not be detected in non-stimulated co-cultures. Inhibition of the mature osteoclast marker integrin-*β*3 has been observed to limit osteoclast migration (54). The observed cell extensions were previously described as a result of osteoclast fission or incomplete cytokinesis, processes that are believed to regulate osteoclastic resorption (55–57). Osteoclast fission has also been described *in vivo* and is suggested to improve osteoclast migration (57). Therefore, we suggest that based on pit shape, integrin-*β*3 expression and the presence of cell extensions, osteoclasts in osteogenically stimulated MC-MSC co-cultures featured a different but more “physiologically relevant” osteoclast phenotype than in non-stimulated co-cultures.

Major issues in the development of *in vitro* models are the difficulty to reproduce these models and to translate results from *in vitro* to *in vivo*. The use of different bovine-derived sera contributes to these issues, because of its xenogeneic origin and its variability between different batches and sources (10, 13, 58). While the replacement of FBS by hPL could improve the biomimicry with physiological bone remodeling, reproducibility issues might still remain unsolved. Characterization and standardization in the preparation of hPL could improve these reproducibility issues (11). Efforts for this standardization and characterization have already been initiated (20, 59, 60). In this study, we therefore used a commercial hPL obtained from >300 donor units whereas a hPL preparation from 200 donor units seems already sufficient for standardization (20). However, like for FBS, batch to batch variability may still exist and this limitation should be taken into consideration when interpreting the results of this study. To support the characterization of bioactive factors in hPL, we aimed at quantifying a panel of bone- and bone remodeling-related factors in hPL. Of importance, these factors were measured in the pure supplements, their concentrations on the cells is dependent on the used concentration of the supplement in the culture medium. We compared the concentrations of the measured factors with the effective concentrations in *in vitro* studies, taking the dilution in the culture medium into account. Although it is expected that the combination of the different hPL components exert the found effects, some factor concentrations stood out. For example, our hPL contained relatively high levels of growth factors like PDGF-BB, bFGF, EGF and VEGF, which likely exceed the growth factor concentrations in FBS (22). However, these growth factors were all below the effective levels derived from *in vitro* studies. Only calcium and IL17 were in the effective range for 10% hPL and 5% hPL, respectively. The relatively high calcium concentration in 10% hPL could improve proteolytic activity in osteoclasts, while a low concentration improves attachment and migration (38). In addition, the IL17 concentrations in 10% and 5% hPL have previously directly induced osteoclastic differentiation of human buffy coat derived monocytes in the absence of osteoblasts (61). To proof whether such factors have had significant effects in this study, inhibition or removal of these factor would need to be performed which was outside the scope of this research. Besides, synergistic effects of factors might also be possible (62). Of note, due to the presence of fibrinogen in hPL, coagulation is often prevented by the addition of heparin. However, heparin supplementation could induce the secretion of inflammatory cytokines in macrophages (31). In addition, heparin has a high affinity to OPG, meaning that it could influence the physiological MSC-osteoblast-osteocyte regulated inhibition of osteoclasts (63). For these reasons, using heparin as anti-coagulant for hPL should always be limited or avoided for MC-MSC co-cultures. Other methods to prevent coagulation, such as mechanical fibrinogen-depletion, could prevent the need for heparin supplementation (64). Although for this study fibrinogen-depleted hPL was used, potential remnants of heparin also might have caused the discrepancy between the OPG concentrations measured with Luminex and ELISA, as heparin could induce coagulation of OPG microspheres in Luminex (41).

With this study, we demonstrated that FBS can be replaced by hPL for osteoclastic differentiation of human MCs. A hPL concentration of 2.5% is already sufficient for homogeneous osteoclastic differentiation, but resorption can be enhanced by increasing the concentration to 5% or 10%. In contrast to FBS, hPL could support both osteoclastic and osteogenic differentiation. The addition of 10% hPL to co-cultures will likely lead to a balance towards formation, while 2.5% will shift the balance towards resorption. Thus, a concentration of 5% hPL is recommended. These findings indicate hPL’s potential for *in vitro* bone remodeling models. The use of hPL could therefore limit the need for FBS, which is currently the common standard for these models (5). Accordingly, this study directly contributes to the reduction, refinement and replacement of animal experiments.

## Data Availability Statement

The original contributions presented in the study are included in the article/**Supplementary Material**. Further inquiries can be directed to the corresponding author.

## Ethics Statement

Ethical review and approval was not required for the study on human participants in accordance with the local legislation and institutional requirements. The patients/participants provided their written informed consent to participate in this study.

## Author Contributions

BW, KI, and SH contributed to conception, methodology and design of the study. BW performed and analysed the experiments. BW wrote the original draft of the manuscript and prepared the figures. KI and SH contributed in the supervision. SH acquired funding for this research. All authors contributed to manuscript revision and approved the submitted version.

## Funding

This work is part of the research program TTW with project number TTW 016.Vidi.188.021, which is (partly) financed by the Netherlands Organization for Scientific Research (NWO).

## Conflict of Interest

The authors declare that the research was conducted in the absence of any commercial or financial relationships that could be construed as a potential conflict of interest.

## Publisher’s Note

All claims expressed in this article are solely those of the authors and do not necessarily represent those of their affiliated organizations, or those of the publisher, the editors and the reviewers. Any product that may be evaluated in this article, or claim that may be made by its manufacturer, is not guaranteed or endorsed by the publisher.
