# Supplementary material for: Human Platelet Lysate as Alternative of Fetal Bovine Serum for Enhanced Human In Vitro Bone Resorption and Remodeling
Source: Front Immunol. 2022 Jun 20;13:915277. doi: 10.3389/fimmu.2022.915277 (PMC9251547; doi:10.3389/fimmu.2022.915277)
Supplement: Supplementary file 1 [file DataSheet_1.pdf]

Supplementary Material

1 Supplementary materials and methods

We investigated the influence of FBS and hPL on osteogenic differentiation and bone-like matrix production in a three-dimensional (3D) MSC mono-culture. The materials and methods used for this part of the study are described below. The results on this part of the study can be found in Figure S4.

1.1 Scaffold fabrication

Bombyx mori L. silkworm cocoons were degummed by boiling them in 0.2 M Na_2CO_3 for 1 h. After drying, silk was dissolved in 9 M LiBr, filtered, and dialyzed against ultra-pure water (UPW) for 36 h using SnakeSkin Dialysis Tubing (11532541, Thermo Fisher Scientific, Breda, The Netherlands). The dialyzed silk fibroin solution was frozen at -80º C and lyophilized for 7 days. Lyophilized silk fibroin was dissolved in hexafluoro-2-propanol at a concentration of 17% (w/v) and casted in scaffold molds containing NaCl granules with a size of 250-300 μm as template for the pores. Molds were covered to improve the silk fibroin blending with the granules. After 3 h, covers were removed from molds, and hexafluoro-2-propanol was allowed to evaporate for 7 days whereafter β-sheets were induced by submerging silk fibroin-salt blocks in 90% MeOH for 30 min. NaCl was dissolved from the scaffolds in ultra-pure water, resulting in porous sponges. These sponges were cut into scaffolds of 3 mm in height and 5 mm in diameter. Scaffolds were sterilized by autoclavation in PBS at 121º C for 20 min.

1.2 Cell culture

Mesenchymal stromal cells (MSCs) were isolated from human bone marrow (Lonza, Walkersville, MD, USA) and characterized for surface markers and multilineage differentiation, as previously described (1). MSCs were frozen at passage 3 with $1.25*10^6$ cells/ml in freezing medium containing fetal bovine serum (FBS, BCBV7611, Sigma-Aldrich, Zwijndrecht, The Netherlands) with 10% DMSO and stored in liquid nitrogen until further use. Before experiments, MSCs were thawed, collected in high glucose DMEM (hg-DMEM, 41966, Thermo Fisher Scientific), seeded at a density of $2.5*10^3$ cells/cm^2 and expanded in expansion medium containing hg-DMEM, 10% FBS (BCBV7611, Sigma-Aldrich), 1% Antibiotic Antimycotic (anti-anti, 15240, Thermo Fisher Scientific), 1% Non-Essential Amino Acids (11140, Thermo Fisher Scientific), and 1 ng/mL basic fibroblastic growth factor (bFGF, 100-18B, PeproTech, London, UK) at 37 ºC and 5% CO_2. After 9 days, cells were detached using 0.25% trypsin-EDTA (25200, Thermo Fisher Scientific) and seeded onto scaffolds at passage 4. Cells were seeded at a density of 10^6 cells per scaffold (N = 4 scaffolds per condition) and seeding was performed dynamically for 6 hours in 50 ml tubes on an orbital shaker at 150 RPM in expansion medium (2). The cell-loaded scaffolds were cultured for 4 weeks at 37 ºC and 5% CO_2 in custom-made spinner flask bioreactors and a rotational speed of 300 RPM. MSCs were stimulated to undergo osteogenic differentiation with osteogenic medium containing lg-DMEM (22320, Thermo Fisher Scientific), 10% FBS (SFBS, Bovogen, East Keilor, Australia) or 10%, 5%, or 2.5% human platelet lyste (hPL, PE20612, PL BioScience, Aachen, Germany), 1% anti-anti, 10 mM β-glycerophosphate (G9422, Sigma-Aldrich), 50 μg/ml ascorbic acid-2-phosphate (A8960, Sigma Aldrich), and 100 nM Dexamethasone (D4902, Sigma-Aldrich)). Medium was refreshed 3 times per week and samples were collected and stored at -80 ºC on day 7.

1.3 Micro-computed tomography (μCT)

Bioreactors were scanned and analyzed with a μCT100 imaging system (Scanco Medical, Brüttisellen, Switzerland) after 4 weeks of culture. Scanning was performed at an isotropic nominal resolution of 17.2 μm, energy level of 45 kVp, intensity of 200 μA, integration time of 300 ms and with twofold frame averaging. To reduce part of the noise, a constrained Gaussian filter was applied with filter support 1 and filter width sigma 0.8 voxel. Filtered images were segmented to detect mineralization at a global threshold of 24% of the maximum grayscale value. Unconnected objects smaller than 30 voxels were removed through component labeling.

1.4 (Immuno)histochemistry

Scaffolds (N = 2) were soaked for 15 minutes in each 5% (w/v) sucrose and 35% (w/v) sucrose in phosphate buffered saline (PBS). Samples were embedded in Tissue Tek® (Sakura, Alphen aan den Rijn, The Netherlands) and quickly frozen with liquid N2. Cryosections were sliced with a thickness of 5 µm. Upon staining, sections were fixed for 10 minutes in 3.7% neutral buffered formaldehyde and washed twice with PBS.

To visualize collagen deposition, sections were stained with Picrosirius Red. Sections were soaked in Weigert's Iron Hematoxylin (HT1079, Sigma-Aldrich) solution for 10 minutes, washed in running tap water for 10 minutes, and stained in 1% w/v Sirius Red (36,554-8, Sigma-Aldrich) in picric acid solution (36011, Sigma-Aldrich) for one hour. Subsequently, sections were washed in two changes of 0.5% acetic acid and dehydrated in one change of 70% and 96% EtOH, three changes of 100% EtOH, and two changes of xylene. Sections were mounted with Entellan (107961 Sigma-Aldrich) and imaged with a bright field microscope (Zeiss Axio Observer Z1, 20x/0.8 Plan-Apochromat objective).

To study osteogenic differentiation, sections were stained with DAPI, CNA35, osteopontin and runt-related transcription factor 2 (RUNX2). Briefly, sections were permeabilized in 0.5% triton X-100 in PBS for 5 min and blocked in 10% normal goat serum in PBS for 30 min. Primary antibodies were incubated overnight at 4 ºC, secondary antibodies were incubated with 0.1 μg/ml DAPI and 1 μmol/mL CNA35-mCherry (3) for 1 h at room temperature. Antibodies are listed in Table S1. Images were acquired with a laser scanning microscope (Leica TCS SP5X, 63x/1.2 HCX PL Apo CS objective). All images were prepared for presentation in Fiji (4).

1.5 DNA quantification

Lyophilized samples (N = 3) were weighted and digested overnight in papain digestion buffer (containing 100 mmol phosphate buffer, 5 mmol L-cystein, 5 mmol EDTA and 140 μg/ml papain (P4762, Sigma-Aldrich)). DNA was quantified using the Qubit Quantification Platform (Q32851, Thermo Fisher Scientific), according to the manufacturer's instructions.

1.6 Alkaline phosphatase activity

Scaffolds (N = 3) were washed in PBS and disintegrated using steel balls and a mini-beadbeater™ (Biospec, Bartlesville, OK, USA) in cell lysis buffer containing 0.2% (v/v) Triton X-100 and 5 mM $MgCl_2$. Alkaline phosphatase (ALP) activity in cell lysates was determined by adding 20 μl of 0.75 M 2-amino-2-methyl-1-propanol (A65182, Sigma-Aldrich) to 80 μl sample in 96-wells assay plates.

Subsequently, 100 μl substrate solution (10 mM p-nitrophenyl-phosphate (71768, Sigma-Aldrich) in 0.75 M 2-amino-2-methyl-1-propanol) was added and wells were incubated at room temperature for 15 minutes. To stop the reaction, 100 μl 0.2 M NaOH was added. Absorbance was measured with a plate reader at 450 nm and these values were converted to ALP activity (converted p-nitrophenyl phosphate in μmol/ml/min) using standard curve absorbance values.

1.7 Receptor activator of nuclear factor κB ligand (RANKL) and osteoprotegerin (OPG) quantification

Secreted RANKL and OPG were quantified in cell supernatants from day 7 of 2 different bioreactors containing 4 scaffolds each (N = 2) with RANKL (ab213841, Abcam, Cambridge, UK) and OPG (EHTNFRSF11B, Thermo Fisher Scientific) enzyme-linked immunosorbent assays (ELISAs) according to the manufacturer's protocols. To measure RANKL, samples were added to anti-human RANKL coated microwells. After 90 min incubation at 37 ºC, samples were replaced by biotinylated antibody solution followed by 60 min incubation at 37 ºC. After thorough washing, avidin-biotin-peroxidase complex (ABC) solution was added and plates were incubated for 30 min at 37 ºC. Wells were again washed and color developing agent was added followed by 15 min incubation in the dark at 37 ºC. To stop the reaction, stop solution was added and absorbance was measured at 450 nm in a plate reader. To measure OPG, samples were added to anti-human OPG coated microwells and incubated for 2.5 h at room temperature with gentle shaking. Wells were subsequently washed, biotinylated antibody solution was added followed by 60 min incubation at room temperature with gentle shaking. After washing, streptavidin-HRP solution was added and incubated in the wells for 45 min with gentle shaking. Wells were subsequently washed and incubated with substrate solution for 30 min in the dark with gentle shaking. The enzymatic reaction was stopped with stop solution and absorbance was measured at 450 nm in a plate reader. All absorbance values were converted to RANKL and OPG concentrations using standard curve absorbance values.

2 Supplementary Tables

The antibodies that were used for immunofluorescent stainings of MC-MSC co-cultures (non-stimulated and osteogenically stimulated) and three-dimensional osteogenically stimulated MSC mono-cultures are listed in Table S1.

Table S1. List of antibodies that were used in this study.

Antigen	Supplier	Catalogue No.	Conjugate	Species	Dilution
RANKL	Abcam	ab45039		Mouse	1:200
OPG	Abcam	ab9986		Rabbit	1:500
Integrin-β3	Biorbyt	orb248939		Mouse	1:200
RUNX2	Abcam	ab23981		Rabbit	1:500
Osteopontin	Thermo Fisher	14-9096-82		Mouse	1:200
Anti-mouse IgG1	Molecular Probes	A21121	Alexa 488	Goat	1:200
Anti-Rabbit IgG	Molecular Probes	A21428	Alexa 555	Goat	1:200
Anti-Rabbit IgG	Molecular Probes	A21244	Alexa 647	Goat	1:200

Abbreviations: runt-related transcription factor 2 (RUNX2), receptor activator of nuclear factor κB ligand (RANKL), osteoprotegerin (OPG).

To explore the protein content of hPL, a total of 21 proteins that have been reported to influence bone resorption, formation or remodeling were quantified using multiplex immunoassays. In addition, calcium and phosphate concentration were quantified as well. The concentrations from these analyses were compared to effective concentrations from *in vitro* experiments reported in literature. The results from these quantifications and the literature research are reported in Table S2.

Table S2. Measured concentrations from hPL characterization experiments and effective concentrations reported in literature.

**Analyte**	**Effect on bone remodeling**	**Effective conc. from *in vitro* experiments**	**Conc. in hPL**	**Unit**
IL1-α	Could enhance osteoclastic differentiation in the presence of RANKL (5,6)<br><br>Suggested to enhance osteoclast survival (7)<br><br>Can induce osteoblast apoptosis and inhibit osteogenesis (8)	10,000<br><br>NR<br><br>500 – 10,000	283.94	pg/ml
IL1-β	Could enhance osteoclastic differentiation in the presence of RANKL (5,6)<br><br>Could inhibit osteogenesis by decreasing RUNX2 expression, can stimulate ALP production and mineralization (9)	NR<br><br>100 – 1,000	207.67	pg/ml
IL4	Can inhibit osteoclastic resorption in a dose dependent manner (10)<br><br>Can inhibit osteoclast formation (11)<br><br>Can inhibit osteogenesis in adipose tissue derived MSCs, which can be counteracted by IL6 (12,13)<br><br>Can inhibit osteoblast proliferation and promote ALP production	10,000 – 100,000<br><br>100 – 10,000<br><br>10,000<br><br>100 – 10,000	32.72	pg/ml
IL6	Could enhance osteoclastic differentiation in co-culture by stimulating RANKL production by co-cultured cells (14)	NR	623.59	pg/ml

	In co- presence of TNF-α, can induce osteoclastic differentiation in absence of RANKL (15)<br><br>Stimulatory and inhibitory effects on osteogenesis/osteoblasts reported, dependent on cell differentiation state (14,16)	50,000<br><br>10,000		
IL10	Could suppress osteoclastic differentiation (17)<br><br>Can promote osteogenic differentiation of bone marrow derived MSCs at low physiological concentrations (18)<br><br>Can inhibit osteogenic differentiation of bone marrow derived MSCs at high pathological concentration (18)	NR<br><br>10 – 1,000<br><br>10,000 - 100,000	196.80	pg/ml
IL17	Different outcomes on osteoclasts reported (19)<br><br>Could directly induce osteoclastic differentiation (20)<br><br>Enhanced proliferation and stimulated osteogenesis. Can induce RANKL and M-CSF expression and osteoclastic differentatiation in co-culture with PBMCs (21)	NA<br><br>10 – 1,000<br><br>20,000 – 50,000	369.86	pg/ml
TNF-α	Could enhance osteoclastic differentiation in the presence of RANKL (6)<br><br>In co- presence of IL-6, can induce osteoclastic differentiation in absence of RANKL (15)<br><br>Could inhibit osteogenesis by decreasing RUNX2 expression, can stimulate ALP production and mineralization (9)	NR<br><br>50,000<br><br>100 – 10,000	236.61	pg/ml

SDF-1α	Hypothesized to recruit osteoclast precursors (22)<br><br>Important role in migration of MSCs, especially in inflammation (23)<br><br>Could support early osteogenic differentiation (24)	~8.526 – 255.8*<br><br>150<br><br>NR	3.292	ng/ml
RANKL	Expressed by a multiple cell types, but typically by osteoblastic cells, required for osteoclast differentiation (25). Multiple concentrations are used to induce osteoclastogenesis *in vitro* (26)	10,000 – 100,000	394.34	pg/ml
OPG	Can prevent RANKL from binding to the RANK receptor on preosteoclasts, inhibits osteoclastogenesis (25,27)	20 – 100	1.6876	ng/ml
Sclerostin	Inhibits bone formation and osteogenesis and could stimulate RANKL secretion by osteocytes, thereby promoting osteoclastogenesis (28)	NR	6.4137	ng/ml
Osteopontin	Instrumental for intrafibrillar mineralization and promotes osteoclast activation (29)<br><br>Stimulates osteoclastogenesis and plays a major role in the formation of sealing zones (30)<br><br>Promotes osteoclast precursor migration (31)	100,000**<br><br>NA<br><br>~600 – 60,000*	2.1524	ng/ml
Dkk-1	Inhibitor for osteogenic differentiation and bone formation. Can inhibit osteoclast induced mineralization by osteoblasts (32)	200	1.86	ng/ml
M-CSF	Can regulate multiple steps of human *in vitro* osteoclastogenesis, including osteoclast precursor proliferation, differentiation, and fusion, and osteoclast resorption (33)	25,000	346.29	pg/ml

GM-CSF	Can suppress osteoclastogenesis in early differentiation stages, but promotes fusion of mature osteoclasts (34)	3,000	381.78	pg/ml
EGF	Stimulatory effect on osteogenic differentiation of dental pulp stem cells, could enhance mineralization (35)<br><br>Could promote osteoblast proliferation and protein expression, but not mineralization (36)<br><br>Might improve osteoclast survival and differentiation through binding to the EFG receptor (37)<br><br>Could promote ALP production and mineralization by MSCs (38)	10<br><br>10<br><br>NA<br><br>50	1.6238	ng/ml
Basic FGF	Inhibitory effect on osteogenic differentiation of dental pulp stem cells (35)<br><br>Might promote osteoblast-like cell differentiation towards osteocyte (39)<br><br>Contradictory results. Likely, proliferative and stemness maintaining effect at lower concentrations (40)<br><br>Could inhibit osteoclast formation when co-cultured with MSC-like cells (41)	10,000<br><br>10,000<br><br>≤ 10,000<br><br>1,000 – 10,000	811.81	pg/ml
VEGF	Could enhance osteoclast survival and resorption (42,43)<br><br>Intracellular but not exogenous inhibits adipogenic differentation and promotes osteogenic differentiation of bone marrow derived MSCs (43)	5,000 – 150,000<br><br>NA	868.60	pg/ml
Fetuin	Could inhibit osteogenic differentiation and mineralization, could induce	4,840,000,000*	297.13	ng/ml

	adipogenic differentiation in bone marrow cultures (44)<br><br>Might inhibit extrafibrillar collagen mineralization (45)	 <br>NR		
Fibronectin	Could inhibit osteoclastogenesis (46,47)<br><br>Could enhance mature osteoclast activity and resorption (46)<br><br>Could enhance osteogenic differentiation and bone-like matrix formation of bone marrow derived MSCs at low coating densities, and inhibit differentiation but promote proliferation at higher coating densities (48)	0.1 – 20**<br><br>20**<br><br>NA	3.2096	μg/ml
PDGF-BB	Can enhance osteoclastogenesis of macrophage-like cells (49)<br><br>Could promote osteogenic differentiation of adipose derived but not bone marrow derived MSCs (50)<br><br>No effect on ALP formation and mineralization by MSCs (38)	20 - 50<br><br>20<br><br>10	8.159	ng/ml
Calcium	Higher proteolytic activity in osteoclasts cultured with high calcium concentration than when cultured with a low concentration. Could improve attachment and migration with low calcium concentrations (51)<br><br>Too high calcium concentrations can inhibit osteoclast activity and from 20 μmol/ml induce osteoclast apoptosis (51,52)<br><br>Could promote proliferation and osteogenic differentiation of MSCs (53)	1.2 (high)<br><br>0.5 (low)<br><br>5 - 20<br><br>7.8	15.49	μmol/ml

Phosphate	Inhibited osteoclastogenesis from bone marrow cultures in co-culture with osteoblast-like cells (54)<br><br>Inhibited osteoclastogenesis of human PBMCs and macrophage-like cells in a dose-dependent response (55)<br><br>Can promote proliferation of human bone marrow derived MSCs (56)<br><br>Can promote migration, osteogenic differentiation and mineralization of human bone marrow derived MSCs (56)	1 – 10<br><br>1.5 – 4<br><br>2 – 10<br><br>4 – 10	1.948	μmol/ml

* Calculated from the molecular weight found in literature

** Concentration used for coating of culture substrate

Abbreviations: human platelet lysate (hPL), concentration (conc.), not reported (NR), not applicable (NA), monocyte (MC), mesenchymal stromal cell (MSC), peripheral blood mononuclear cell (PBMC), runt-related transcription factor 2 (RUNX2), alkaline phosphatase (ALP), interleukin (IL), tumor necrosis factor (TNF), stromal derived factor (SDF), granulocyte-macrophage colony-stimulating factor (GM-CSF), macrophage colony-stimulating factor (M-CSF), receptor activator of nuclear factor κB ligand (RANKL), osteoprotegerin (OPG), Dickkopf WNT Signaling Pathway Inhibitor 1 (Dkk-1), epidermal growth factor (EGF), basic fibroblastic growth factor (bFGF), vascular endothelial growth factor (VEGF), platelet derived growth factor-BB (PDGF-BB).

3 Supplementary Figures

To visualize the non-resorbed surface, osteo assay wells were stained with a modified Von Kossa. To capture the entire well, tile scans were made with a bright field microscope. Tile scans were stitched with Zen Blue software (version 3.1, Zeiss, Breda, The Netherlands). To enable segmentation and resorption quantification, scratches that were introduced by mechanical cell removal in co-cultures were manually masked whereafter image contrast was increased using Fiji (4). A clipping mask was created in Illustrator (Adobe Inc., San Jose, CA, USA) to remove the edges of the wells. Segmentation was performed in MATLAB (version 2019b, The MathWorks Inc., Natrick, MA, USA), using Otsu's method for binarization with global thresholding, where the threshold was kept constant throughout the entire image (Figure S1) (57).

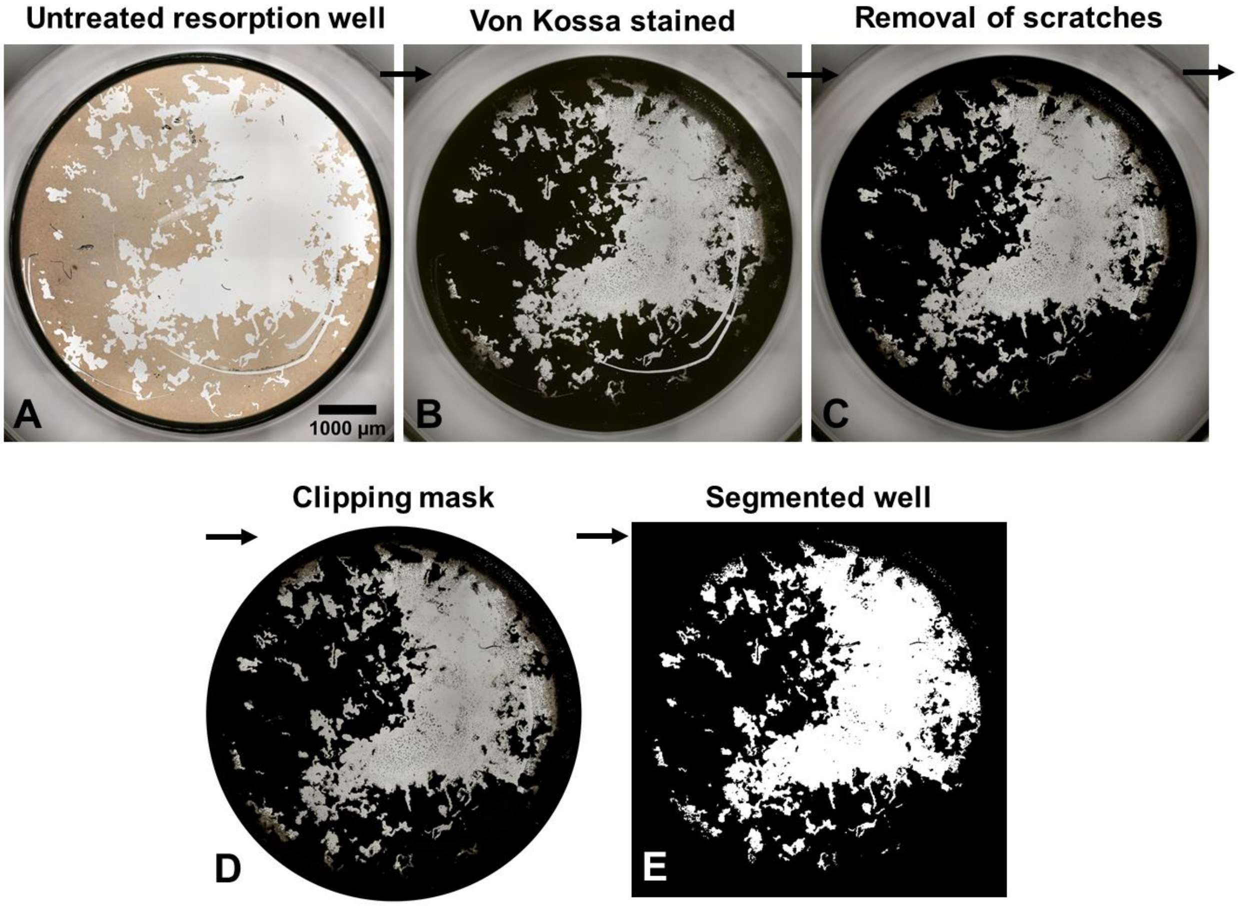

Figure S1. Workflow osteo assay wells from raw data to image segmentation. Decellularized resorption wells (**A**) were stained with Von Kossa (**B**). Scratches were manually masked (**C**) and a clipping mask was used to remove the edges of the well (**D**). Lastly, images were segmented such that the resorbed surface could be quantified (**E**).

As osteoclasts have a life-span of approximately 2-3 weeks (58,59), culture photographs were taken on day 18/21 of MC mono-cultures (Figure S2). By day 18, MCs have likely differentiated into mature osteoclasts and apoptosis might not yet have taken place.

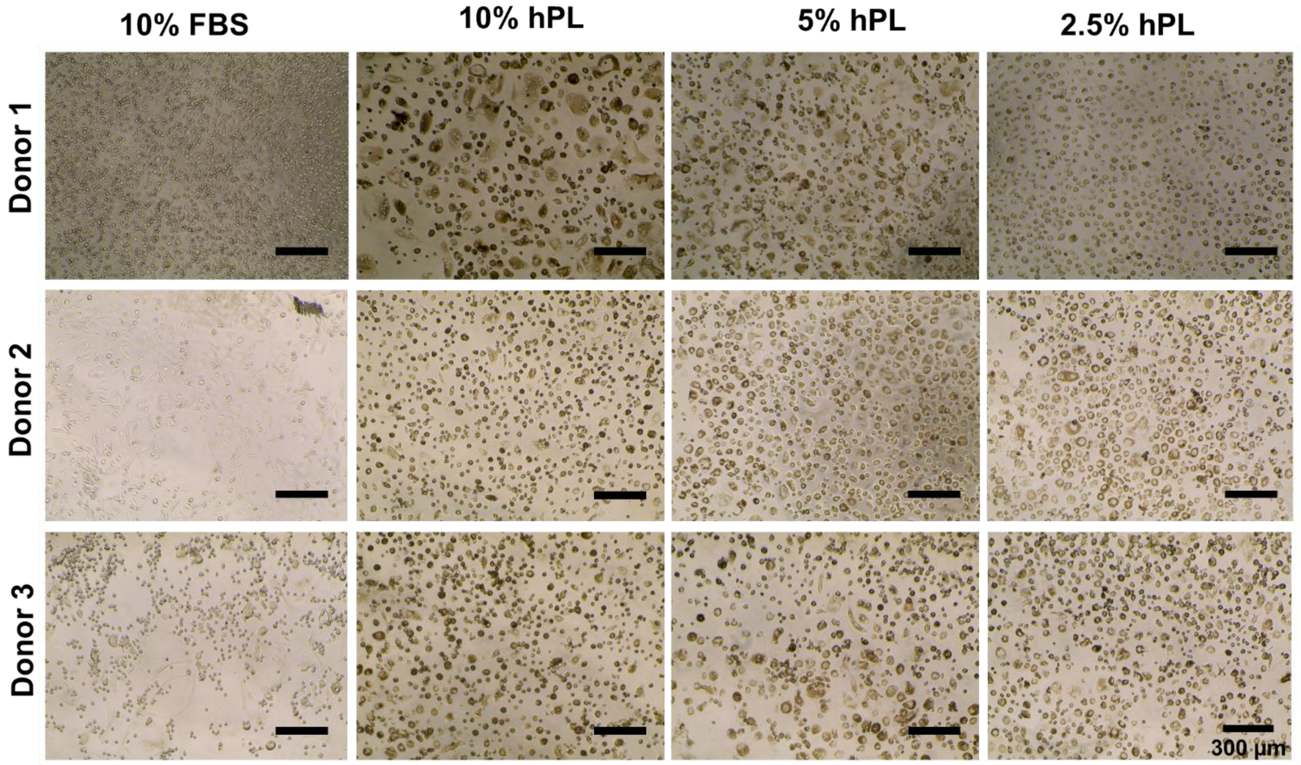

Figure S2. Micrographs of all MC donors on day 18 in mono-cultures. Clear differences can be observed between MCs cultured with FBS and hPL, indicating a heterogeneous cell population in MCs cultured with FBS. No clear differences were observed between donors, with only in donor 1 a concentration dependent size difference in MCs cultured with hPL. Abbreviations: fetal bovine serum (FBS), human platelet lysate (hPL), monocyte (MC).

To check whether osteogenically stimulated MC-MSC co-cultures showed differences in RANKL and OPG expression, cells were stained for these proteins (Figure S3).

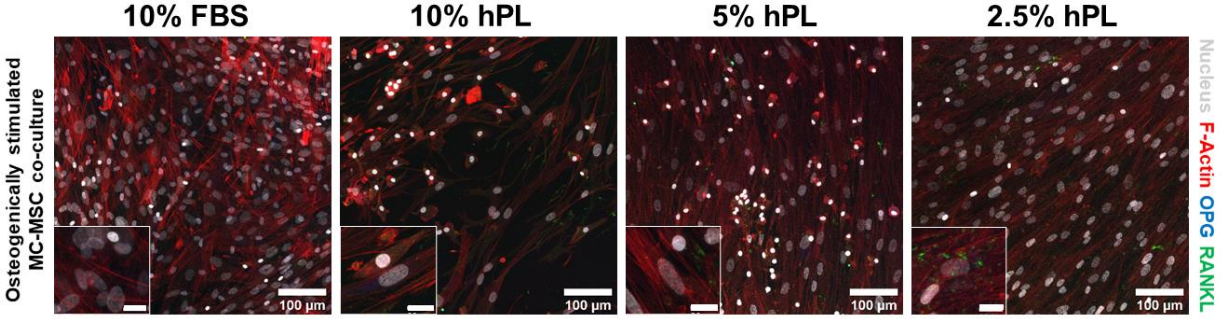

Figure S3. Micrographs of osteogenically stimulated MC-MSC co-cultures, stained for F-Actin (red), the nucleus (gray), OPG (blue) and RANKL (green). It is expected that OPG and RANKL also played a role in the inhibition of osteoclastic differentiation in osteogenically stimulated MC-MSC co-cultures, although no clear differences between groups were found after immunocytochemical staining of OPG and RANKL. Scale bar in insert is 20 μm. Abbreviations: fetal bovine serum (FBS), human platelet lysate (hPL), monocyte (MC), mesenchymal stromal cell (MSC), receptor activator of nuclear factor κB ligand (RANKL), osteoprotegerin (OPG).

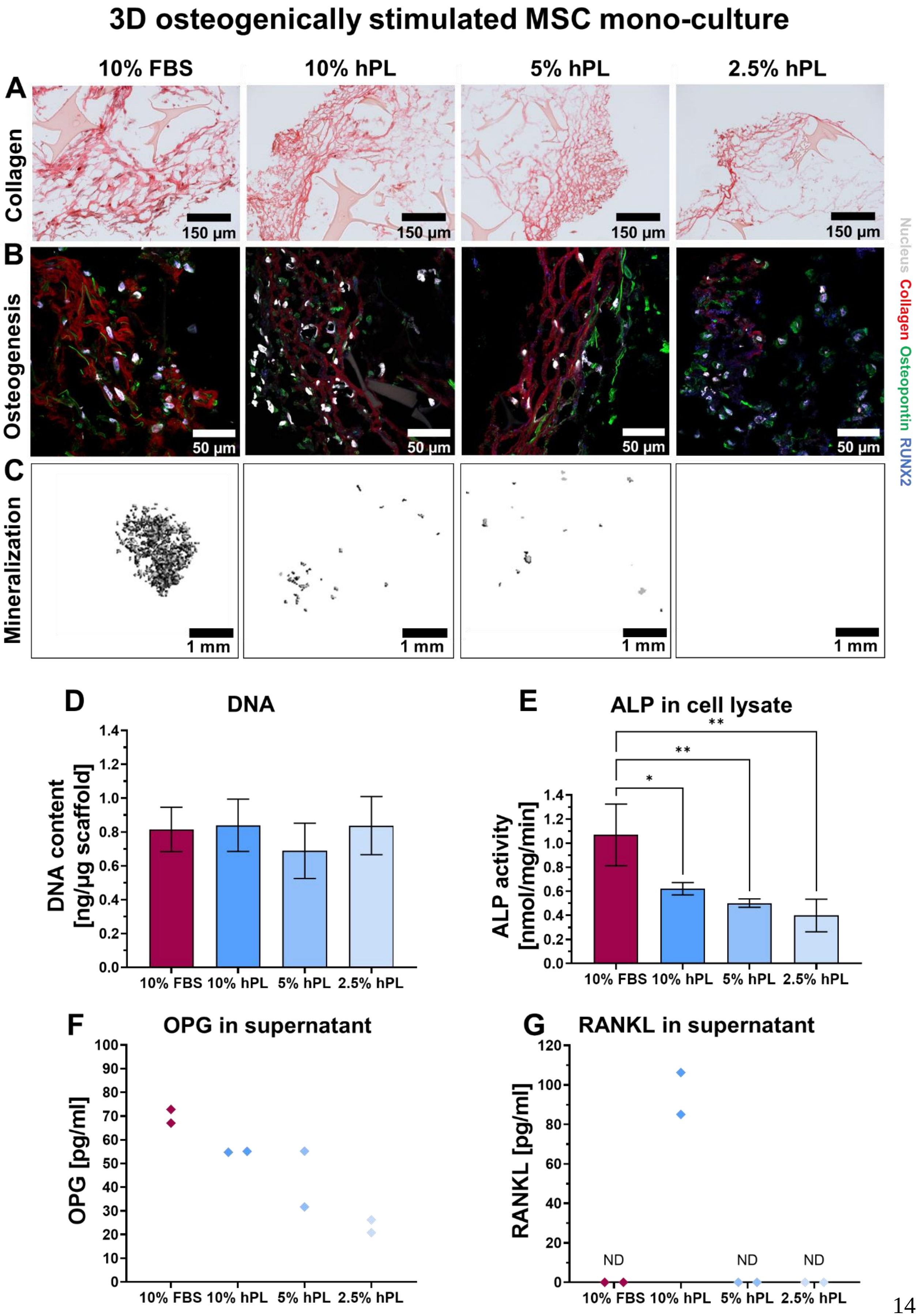
3D osteogenically stimulated MSC mono-culture
10% FBS
10% hPL
5% hPL
2.5% hPL
A
Collagen
150 µm
B
Osteogenesis
50 µm
Nucleus Collagen Osteopontin RUNX2
C
Mineralization
1 mm
D
DNA
DNA content [ng/µg scaffold]
E
ALP in cell lysate
ALP activity [nmol/mg/min]
F
OPG in supernatant
OPG [pg/ml]
G
RANKL in supernatant
RANKL [pg/ml]
ND

Figure S4. Results from 3D osteogenically stimulated MSC mono-cultures indicating most osteogenic differentiation and bone-like matrix formation in MSCs cultured with FBS. (**A**) Picrosirius red staining indicating collagen formation in osteogenically stimulated 3D MSC monocultures after 4 weeks culture, most collagen seemed present in constructs cultured with 10% FBS and 10% hPL. (**B**) Staining for osteogenic markers indicating osteogenic differentiation of 3D MSC monocultures after 4 weeks culture in all conditions. Sections were stained for cell nuclei (gray), collagen (red), osteopontin (green) and RUNX2 (blue). (**C**) Micro-computed tomography scans of constructs, indicating most mineralization in constructs cultured with FBS. No mineralization was found in constructs cultured with 2.5% hPL. (**D**) DNA quantification in cultured constructs, no clear differences were found between different conditions. (**E**) ALP activity quantification revealed most ALP in lysates of constructs cultured with 10% FBS, $p<0.05$ (one-way ANOVA and Turkey's post hoc tests) (**F**) OPG quantification in cell supernatants of 2 bioreactors (containing 4 tissue constructs each) revealed most OPG in constructs cultured with 10% FBS. (**G**) RANKL was only detected in constructs cultured with 10% hPL. (*$p<0.05$, **$p<0.01$) Abbreviations: fetal bovine serum (FBS), human platelet lysate (hPL), monocytes (MCs), mesenchymal stromal cells (MSCs), runt-related transcription factor 2 (RUNX2), alkaline phosphatase (ALP), osteoprotegerin (OPG), receptor activator of nuclear factor κB ligand (RANKL), not detected (ND).

4 Supplementary References

1. Hofmann S, Hagenmüller H, Koch AM, Müller R, Vunjak-Novakovic G, Kaplan DL, Merkle HP, Meinel L. Control of in vitro tissue-engineered bone-like structures using human mesenchymal stem cells and porous silk scaffolds. *Biomaterials* (2007) **28**:1152–1162. doi: 10.1016/j.biomaterials.2006.10.019

2. Melke J, Zhao F, Ito K, Hofmann S. Orbital seeding of mesenchymal stromal cells increases osteogenic differentiation and bone - like tissue formation. *J Orthop Res* (2019) **2020**:1–10. doi: 10.1002/jor.24583

3. Aper SJA, van Spreeuwel ACC, van Turnhout MC, van der Linden AJ, Pieters PA, van der Zon NLL, de La Rambelje SL, Bouten CVC, Merkx M. Colorful protein-based fluorescent probes for collagen imaging. *PLoS one* (2014) **9**: doi: 10.1371/journal.pone.0114983

4. Schindelin J, Arganda-Carreras I, Frise E, Kaynig V, Longair M, Pietzsch T, Preibisch S, Rueden C, Saalfeld S, Schmid B, et al. Fiji: An open-source platform for biological-image analysis. *Nature Methods* (2012) **9**:676–682. doi: 10.1038/nmeth.2019

5. Lee YM, Fujikado N, Manaka H, Yasuda H, Iwakura Y. IL-1 plays an important role in the bone metabolism under physiological conditions. *International Immunology* (2010) **22**:805–816. doi: 10.1093/intimm/dxq431

6. Osta B, Benedetti G, Miossec P. Classical and paradoxical effects of TNF-α on bone homeostasis. *Frontiers in Immunology* (2014) **5**: doi: 10.3389/fimmu.2014.00048

7. Soysa NS, Alles N. Positive and negative regulators of osteoclast apoptosis. *Bone Reports* (2019) **11**: doi: 10.1016/j.bonr.2019.100225

8. Guo C, Yang XG, Wang F, Ma XY. IL-1 induces apoptosis and inhibits the osteoblast differentiation of MC3T3-E1 cells through the JNK and p38 MAPK pathways. *International Journal of Molecular Medicine* (2016) **38**:319–327. doi: 10.3892/ijmm.2016.2606

9. Ding J, Ghali O, Lencel P, Broux O, Chauveau C, Devedjian JC, Hardouin P, Magne D. TNF-α and IL-1β inhibit RUNX2 and collagen expression but increase alkaline phosphatase activity and mineralization in human mesenchymal stem cells. *Life Sciences* (2009) **84**:499–504. doi: 10.1016/j.lfs.2009.01.013

10. Mangashetti LS, Khapli SM, Wani MR. IL-4 Inhibits Bone-Resorbing Activity of Mature Osteoclasts by Affecting NF-κB and Ca 2+ Signaling . *The Journal of Immunology* (2005) **175**:917–925. doi: 10.4049/jimmunol.175.2.917

11. Bendixen AC, Shevde NK, Dienger KM, Willson TM, Funk CD, Pike JW. IL-4 inhibits osteoclast formation through a direct action on osteoclast precursors via peroxisome proliferator-activated receptor gamma 1. *PNAS* (2001) **98**:2443–2448. doi: 10.1073/pnas.041493198

12. Bastidas-Coral AP, Bakker AD, Zandieh-Doulabi B, Kleverlaan CJ, Bravenboer N, Forouzanfar T, Klein-Nulend J. Cytokines TNF-α, IL-6, IL-17F, and IL-4 Differentially Affect Osteogenic Differentiation of Human Adipose Stem Cells. *Stem Cells International* (2016) **2016**: doi: 10.1155/2016/1318256

13. Bastidas-Coral AP, Hogervorst JMA, Forouzanfar T, Kleverlaan CJ, Koolwijk P, Klein-Nulend J, Bakker AD. IL-6 counteracts the inhibitory effect of IL-4 on osteogenic differentiation of human adipose stem cells. *Journal of Cellular Physiology* (2019) **234**:20520–20532. doi: 10.1002/jcp.28652

14. Takeuchi T, Yoshida H, Tanaka S. Role of interleukin-6 in bone destruction and bone repair in rheumatoid arthritis. *Autoimmunity Reviews* (2021) **20**: doi: 10.1016/j.autrev.2021.102884

15. O'Brien W, Fissel BM, Maeda Y, Yan J, Ge X, Gravallese EM, Aliprantis AO, Charles JF. RANK-Independent Osteoclast Formation and Bone Erosion in Inflammatory Arthritis. *Arthritis and Rheumatology* (2016) **68**:2889–2900. doi: 10.1002/art.39837

16. Li Y, Bäckesjö CM, Haldosén LA, Lindgren U. IL-6 receptor expression and IL-6 effects change during osteoblast differentiation. *Cytokine* (2008) **43**:165–173. doi: 10.1016/j.cyto.2008.05.007

17. Zhang Q, Chen B, Yan F, Guo J, Zhu X, Ma S, Yang W. Interleukin-10 inhibits bone resorption: A potential therapeutic strategy in periodontitis and other bone loss diseases. *BioMed Research International* (2014) **2014**: doi: 10.1155/2014/284836

18. Chen E, Liu G, Zhou X, Zhang W, Wang C, Hu D, Xue D, Pan Z. Concentration-dependent, dual roles of IL-10 in the osteogenesis of human BMSCs via P38/MAPK and NF-kB signaling pathways. *FASEB Journal* (2018) **32**:4917–4929. doi: 10.1096/fj.201701256RRR

19. Lee Y. The role of interleukin-17 in bone metabolism and inflammatory skeletal diseases. *BMB Reports* (2013) **46**:479–483. doi: 10.5483/BMBRep.2013.46.10.141

20. Yago T, Nanke Y, Ichikawa N, Kobashigawa T, Mogi M, Kamatani N, Kotake S. IL-17 induces osteoclastogenesis from human monocytes alone in the absence of osteoblasts, which is potently inhibited by anti-TNF-α antibody: A novel mechanism of osteoclastogenesis by IL-17. *Journal of Cellular Biochemistry* (2009) **108**:947–955. doi: 10.1002/jcb.22326

21. Huang H, Kim HJ, Chang EJ, Lee ZH, Hwang SJ, Kim HM, Lee Y, Kim HH. IL-17 stimulates the proliferation and differentiation of human mesenchymal stem cells: Implications for bone remodeling. *Cell Death and Differentiation* (2009) **16**:1332–1343. doi: 10.1038/cdd.2009.74

22. Yu X, Huang Y, Collin-Osdoby P, Osdoby P. Stromal Cell-Derived Factor-1 (SDF-1) Recruits Osteoclast Precursors by Inducing Chemotaxis, Matrix Metalloproteinase-9 (MMP-9) Activity, and Collagen Transmigration. (2003). 1404–1418 p. doi: 10.1359/jbmr.2003.18.8.1404

23. Ponte AL, Marais E, Gallay N, Langonné A, Delorme B, Hérault O, Charbord P, Domenech J. The In Vitro Migration Capacity of Human Bone Marrow Mesenchymal Stem Cells: Comparison of Chemokine and Growth Factor Chemotactic Activities. *STEM CELLS* (2007) **25**:1737–1745. doi: 10.1634/stemcells.2007-0054

24. Gilbert W, Bragg R, Elmansi AM, McGee-Lawrence ME, Isales CM, Hamrick MW, Hill WD, Fulzele S. Stromal cell-derived factor-1 (CXCL12) and its role in bone and muscle biology. *Cytokine* (2019) **123**: doi: 10.1016/j.cyto.2019.154783

25. Boyce BF, Xing L. Functions of RANKL/RANK/OPG in bone modeling and remodeling. *Arch Biochem Biophys* (2008) **473**:139–146. doi: 10.1016/j.abb.2008.03.018

26. Remmers SJA, de Wildt BWM, Vis MAM, Spaander ESR, de Vries RBM, Ito K, Hofmann S. Osteoblast-osteoclast co-cultures: A systematic review and map of available literature. *PLOS ONE* (2021) **16**:e0257724. doi: 10.1371/journal.pone.0257724

27. Fu YX, Gu JH, Zhang YR, Tong XS, Zhao HY, Yuan Y, Liu XZ, Bian JC, Liu ZP. Osteoprotegerin influences the bone resorption activity of osteoclasts. *International Journal of Molecular Medicine* (2013) **31**:1411–1417. doi: 10.3892/ijmm.2013.1329

28. Suen PK, Qin L. Sclerostin, an emerging therapeutic target for treating osteoporosis and osteoporotic fracture: A general review. *Journal of Orthopaedic Translation* (2016) **4**:1–13. doi: 10.1016/j.jot.2015.08.004

29. Rodriguez D, Thula-Mata T, Toro E, Yeh Y, Holt C, Holliday L, Gower L. Multifunctional role of osteopontin in directing intrafibrillar mineralization of collagen and activation of osteoclasts. *Acta Biomater* (2014) **10**: doi: 10.1111/j.1746-1561.2010.00581.x.Are

30. Singh A, Gill G, Kaur H, Amhmed M, Jakhu H. Role of osteopontin in bone remodeling and orthodontic tooth movement: a review. *Progress in Orthodontics* (2018) **19**: doi: 10.1186/s40510-018-0216-2

31. Terai K, Takano-Yamamoto T, Ohba Y, Hiura K, Sugimoto M, Sato M, Kawahata H, Inaguma N, Kitamura Y, Nomura S. Role of Osteopontin in Bone Remodeling Caused by Mechanical Stress. (1999).

32. Ota K, Quint P, Ruan M, Pederson L, Westendorf JJ, Khosla S, Oursler MJ. TGF-β induces Wnt10b in osteoclasts from female mice to enhance coupling to osteoblasts. *Endocrinology* (2013) **154**:3745–3752. doi: 10.1210/en.2013-1272

33. Hodge JM, Kirkland MA, Nicholson GC. Multiple roles of M-CSF in human osteoclastogenesis. *Journal of Cellular Biochemistry* (2007) **102**:759–768. doi: 10.1002/jcb.21331

34. Lee MS, Kim HS, Yeon J-T, Choi S-W, Chun CH, Kwak HB, Oh J. GM-CSF Regulates Fusion of Mononuclear Osteoclasts into Bone-Resorbing Osteoclasts by Activating the

Ras/ERK Pathway. *The Journal of Immunology* (2009) **183**:3390–3399. doi: 10.4049/jimmunol.0804314

35. del Angel-Mosqueda C, Gutiérrez-Puente Y, López-Lozano AP, Romero-Zavaleta RE, Mendiola-Jiménez A, Medina-De la Garza CE, Márquez-M M, de la Garza-Ramos MA. Epidermal growth factor enhances osteogenic differentiation of dental pulp stem cells in vitro. *Head and Face Medicine* (2015) **11**: doi: 10.1186/s13005-015-0086-5

36. Laflamme C, Curt S, Rouabhia M. Epidermal growth factor and bone morphogenetic proteins upregulate osteoblast proliferation and osteoblastic markers and inhibit bone nodule formation. *Archives of Oral Biology* (2010) **55**:689–701. doi: 10.1016/j.archoralbio.2010.06.010

37. Yi T, Lee HL, Cha JH, Ko S il, Kim HJ, Shin HI, Woo KM, Ryoo HM, Kim GS, Baek JH. Epidermal growth factor receptor regulates osteoclast differentiation and survival through cross-talking with RANK signaling. *Journal of Cellular Physiology* (2008) **217**:409–422. doi: 10.1002/jcp.21511

38. Irina Kratchmarova, Blagoy Blagoev, Mandana Haack-Sorensen, Moustapha Kassem, Matthias Mann. Mechanism of Divergent Growth Factor Effects in Mesenchymal Stem Cell Differentiation. *Science (1979)* (2005) **308**:1472–1477. doi: 10.1126/science.1108408

39. Gupta RR, Yoo DJ, Hebert C, Niger C, Stains JP. Induction of an osteocyte-like phenotype by fibroblast growth factor-2. *Biochemical and Biophysical Research Communications* (2010) **402**:258–264. doi: 10.1016/j.bbrc.2010.10.011

40. Novais A, Chatzopoulou E, Chaussain C, Gorin C. The potential of fgf-2 in craniofacial bone tissue engineering: A review. *Cells* (2021) **10**: doi: 10.3390/cells10040932

41. Jim E, Shuto T, Ikebe T, Jincushi S, Hirata M, Koca T. Basic Fibroblast Growth Factor Inhibits Osteoclast-Like Cell Formation. (1996). 395–402 p.

42. Yang Q, McHugh KP, Patntirapong S, Gu X, Wunderlich L, Hauschka P v. VEGF enhancement of osteoclast survival and bone resorption involves VEGF receptor-2 signaling and β3-integrin. *Matrix Biology* (2008) **27**:589–599. doi: 10.1016/j.matbio.2008.06.005

43. Liu Y, Berendsen AD, Jia S, Lotinun S, Baron R, Ferrara N, Olsen BR. Intracellular VEGF regulates the balance between osteoblast and adipocyte differentiation. *Journal of Clinical Investigation* (2012) **122**:3101–3113. doi: 10.1172/JCI61209

44. Binkert C, Demetriou M, Sukhu B, Szweras M, Tenenbaum HC, Dennis JW. Regulation of Osteogenesis by Fetuin. *The Journal or Biological Chemistry* (1999) **274**:28514–28520. doi: 10.1074/jbc.274.40.28514

45. Brylka L, Jahnen-Dechent W. The role of fetuin-A in physiological and pathological mineralization. *Calcified Tissue International* (2013) **93**:355–364. doi: 10.1007/s00223-012-9690-6

46. Gramoun A, Azizi N, Sodek J, Heersche JNM, Nakchbandi I, Manolson MF. Fibronectin inhibits osteoclastogenesis while enhancing osteoclast activity via nitric oxide and interleukin-1β-mediated signaling pathways. *Journal of Cellular Biochemistry* (2010) **111**:1020–1034. doi: 10.1002/jcb.22791

47. Seiji Goda, Hiroshi Hayashi, Yosuke Ujii, Osamu Takeuchi, Reiko Komasa, Eisuke Domae, Kazuyo Yamamoto, Naoyuki Matsumoto, Takashi Ikeo. Fibronectin inhibited RANKL-induced differentiation into osteoclast. *J Oral Tissue Engin* (2014) **11**:227–233. doi: 10.11223/jarde.11.227

48. Faia-Torres AB, Goren T, Ihalainen TO, Guimond-Lischer S, Charnley M, Rottmar M, Maniura-Weber K, Spencer ND, Reis RL, Textor M, et al. Regulation of human mesenchymal stem cell osteogenesis by specific surface density of fibronectin: A gradient study. *ACS Applied Materials and Interfaces* (2015) **7**:2367–2375. doi: 10.1021/am506951c

49. Li D qi, Wan Q long, Pathak JL, Li Z bing. Platelet-derived growth factor BB enhances osteoclast formation and osteoclast precursor cell chemotaxis. *Journal of Bone and Mineral Metabolism* (2017) **35**:355–365. doi: 10.1007/s00774-016-0773-8

50. Hung BP, Hutton DL, Kozielski KL, Bishop CJ, Naved B, Green JJ, Caplan AI, Gimble JM, Dorafshar AH, Grayson WL. Platelet-Derived Growth Factor BB Enhances Osteogenesis of Adipose-Derived but Not Bone Marrow-Derived Mesenchymal Stromal/Stem Cells. *Stem Cells* (2015) **33**:2773–2784. doi: 10.1002/stem.2060

51. Xiang B, Liu Y, Zhao W, Zhao H, Yu H. Extracellular calcium regulates the adhesion and migration of osteoclasts via integrin αvβ 3/Rho A/Cytoskeleton signaling. *Cell Biology International* (2019) **43**:1125–1136. doi: 10.1002/cbin.11033

52. Lorget F, Kamel S, Mentaverri R, Wattel A, Naassila M, Maamer M, Brazier M. High extracellular calcium concentrations directly stimulate osteoclast apoptosis. *Biochemical and Biophysical Research Communications* (2000) **268**:899–903. doi: 10.1006/bbrc.2000.2229

53. Barradas AMC, Fernandes HAM, Groen N, Chai YC, Schrooten J, van de Peppel J, van Leeuwen JPTM, van Blitterswijk CA, de Boer J. A calcium-induced signaling cascade leading to osteogenic differentiation of human bone marrow-derived mesenchymal stromal cells. *Biomaterials* (2012) **33**:3205–3215. doi: 10.1016/j.biomaterials.2012.01.020

54. Takeyama S, Yoshimura Y, Deyama Y, Sugawara Y, Fukuda H, Matsumoto A. Phosphate decreases osteoclastogenesis in coculture of osteoblast and bone marrow. *Biochemical and Biophysical Research Communications* (2001) **282**:798–802. doi: 10.1006/bbrc.2001.4652

55. Mozar A, Haren N, Chasseraud M, Louvet L, Mazière C, Wattel A, Mentaverri R, Morlière P, Kamel S, Brazier M, et al. High extracellular inorganic phosphate

concentration inhibits RANK-RANKL signaling in osteoclast-like cells. *Journal of Cellular Physiology* (2008) **215**:47–54. doi: 10.1002/jcp.21283

56. Lin H, Zhou Y, Lei Q, Lin D, Chen J, Wu C. Effect of inorganic phosphate on migration and osteogenic differentiation of bone marrow mesenchymal stem cells. *BMC Developmental Biology* (2021) **21**: doi: 10.1186/s12861-020-00229-x

57. Otsu N. A Threshold Selection Method from Gray-Level Histograms. *IEEE Transactions on Systems, Man, and Cybernetics* (1979) **9**:62–66. doi: 10.1109/TSMC.1979.4310076

58. Weinstein RS, Manolagas SC. Apoptosis and Osteoporosis. *Am J Med* (2000) **108**:153–164. doi: 10.1016/s0002-9343(99)00420-9

59. Manolagas SC. Birth and Death of Bone Cells: Basic Regulatory Mechanisms and Implications for the Pathogenesis and Treatment of Osteoporosis. *Endocrine Reviews* (2000) **21**:115–137. doi: 10.1210/edrv.21.2.0395
